# Neural Network-Based Composite Risk Scoring for Stratification of Fecal Immunochemical Test-Positive Patients in Colorectal Cancer Screening: Findings from South-West Oltenia

**DOI:** 10.3390/cancers17111868

**Published:** 2025-06-02

**Authors:** Alexandra-Georgiana Bocioagă, Carmen-Nicoleta Oancea, Dumitru Rădulescu, Bogdan Silviu Ungureanu, Vlad Florin Iovănescu, Dan Nicolae Florescu, Irina-Paula Doica, Victor-Mihai Sacerdoțianu, Liliana Streba, Tudorel Ciurea, Dan-Ionuț Gheonea

**Affiliations:** 1 Research Center of Gastroenterology and Hepatology, University of Medicine and Pharmacy Craiova, 200349 Craiova, Romania; alexandra.bocioaga@umfcv.ro (A.-G.B.); bogdan.ungureanu@umfcv.ro (B.S.U.); vlad.iovanescu@umfcv.ro (V.F.I.); dan.florescu@umfcv.ro (D.N.F.); irina.doica@umfcv.ro (I.-P.D.); mihai.sacerdotianu@umfcv.ro (V.-M.S.); tudorel.ciurea@umfcv.ro (T.C.); dan.gheonea@umfcv.ro (D.-I.G.); 2 Department of Biochemistry, University of Medicine and Pharmacy of Craiova, 200349 Craiova, Romania; carmen.oancea@umfcv.ro; 3 Department of Community Projects, University of Medicine and Pharmacy of Craiova, 200349 Craiova, Romania; 4 Department of Surgery, University of Medicine and Pharmacy of Craiova, 200349 Craiova, Romania; dumitru.radulescu@umfcv.ro; 5 Department of Gastroenterology, University of Medicine and Pharmacy of Craiova, 200349 Craiova, Romania; 6 Department of Oncology, University of Medicine and Pharmacy of Craiova, 200349 Craiova, Romania

**Keywords:** colorectal cancer, screening, risk stratification, artificial intelligence

## Abstract

**Background**: Colorectal cancer (CRC) remains a major cause of cancer-related mortality worldwide, underscoring the need for more efficient and resource-conscious screening strategies. **Methods**: We screened 51,437 individuals (50–74 y) in South-West Oltenia, Romania, with FIT values of ≥20 µg Hb/g. Of the 2825 FIT-positive individuals, 1550 completed colonoscopy, and we recorded their age, sex, residence, education, comorbidities, medications, and FIT values. After imputing < 8% missing data via multiple imputation, we reduced dimensionality with an autoencoder (ReLU, dropout 0.5, L2, 100 epochs, batch 32) and applied K-Means clustering (k = 5). The following are examples of actionable clusters: Cluster 0 (“High-FIT malignant”): FIT > 200 µg/g, age > 65, diabetes; Cluster 2 (“Low-risk mixed”): FIT 100–199 µg/g, age < 60, no comorbidities; Cluster 3 (“Intermediate-risk older”): FIT 150–200 µg/g, ≥3 comorbidities, rural. Cluster labels were then predicted by a feed-forward neural network (64–32 neurons, dropout 0.6) and validated via 5-fold cross-validation plus a temporal hold-out. **Results**: Five distinct patient clusters were identified, enabling the development of a composite risk score. Notably, Cluster 0, characterized by elevated FIT levels, exhibited a malignancy rate of 50.91%, while the overall CRC diagnostic rate among colonoscoped patients was approximately 13.87%. This stratification model enhances the diagnostic yield by prioritizing high-risk patients for urgent colonoscopy and sparing low-risk individuals from unnecessary invasive procedures. **Conclusions**: The AI-driven composite risk score offers a refined framework for CRC risk stratification and optimized resource allocation. Its implementation can lead to earlier detection of advanced lesions, thereby improving patient outcomes. Further external validation on independent cohorts and regions is essential to confirm its broad utility, with potential future integration of additional biomarkers (e.g., genetic or omics-based indicators) to further enhance predictive accuracy.

## Simple Summary

Colorectal cancer is a major cause of death, yet screening resources are limited. We offered a stool blood test called the fecal immunochemical test to more than 51,000 adults in South-West Oltenia, Romania. Of the 2825 whose test showed hidden blood, 1550 agreed to a follow-up colonoscopy. Using artificial intelligence, we split these individuals into five clear groups. In the highest-risk group about half (51%) already had cancer, while no cancers were found in the lowest-risk group. We then turned these patterns into two easy risk scores: a full version that uses age, test level, other ongoing illnesses, medicines and social factors, and a shorter version that needs only age, test level and other illnesses. The full score spotted every cancer while sparing most low-risk people from colonoscopy. By matching medical effort to true need, our approach can make bowel-cancer screening both cheaper and more lifesaving.

## 1. Introduction

Colorectal cancer (CRC) remains one of the leading causes of morbidity and mortality worldwide—it is the third most common cancer and the second leading cause of cancer-related death globally [1]. Epidemiological projections are alarming: the global incidence of CRC is estimated to increase by roughly 80% by 2040 [1]. Moreover, the highest mortality rates are observed in Central and Eastern Europe, highlighting significant disparities in the implementation of preventive measures [1,2].

While CRC typically affects individuals over 50 years of age, recent evidence indicates a notable rise in incidence among younger populations. Both European and international studies have documented increasing trends in CRC among adults under 50, with annual growth rates of up to 7–8% in this cohort [3,4]. This trend towards an earlier onset of CRC has prompted revisions to screening guidelines in several countries—for instance, in the United States, the recommended starting age for screening has been lowered from 50 to 45 years [5]—emphasizing the importance of early detection and the need to adapt prevention strategies to current epidemiological trends.

Effective CRC screening has been shown to significantly reduce mortality by enabling the detection of precancerous lesions and early-stage cancers, where treatment is considerably more effective [2,6]. The fecal immunochemical test (FIT) currently serves as the cornerstone of population-based screening programs in many countries, due to its accessibility, low cost, and robust diagnostic performance in detecting occult blood [6]. However, individuals with a positive FIT require diagnostic colonoscopy for confirmation and potential polypectomy, which poses a major challenge for healthcare systems. Limited colonoscopy capacity for all FIT-positive patients, combined with patient reluctance or difficulties in accessing these services, can result in diagnostic delays or even lost cases [7].

Underserved populations—such as those residing in rural areas, individuals with lower socio-economic status, or those with limited access to healthcare—face additional barriers throughout the screening process. Participation rates in CRC screening programs are often suboptimal in these vulnerable groups, contributing to late-stage cancer detection and poorer outcomes [8,9]. For example, in 2021, Eastern European countries like Romania reported screening rates below 5% among eligible individuals, compared to rates exceeding 70% in Western European nations with well-organized screening programs [9].

Furthermore, a universal screening approach that tests all individuals above a specific age fails to capture the heterogeneity of individual risk; patients of the same age may have vastly different risk profiles based on genetic predisposition, lifestyle, or comorbid conditions. Hence, in recent years, there has been a growing consensus regarding the need for a personalized approach to CRC screening through risk stratification. The basic premise is to tailor the screening intensity and the urgency of intervention according to each individual’s likelihood of developing advanced lesions, thus optimizing the benefit-to-risk ratio and resource utilization [10].

In this context, identifying additional risk parameters (whether clinical, demographic, or environmental) and incorporating them into a comprehensive predictive model is essential for guiding the effective triage of FIT-positive patients. Prior research has demonstrated that simpler predictive models, such as logistic regression, can perform adequately when input variables are limited, illustrating the practical utility of such approaches in routine clinical contexts [11]. Moreover, recent advancements have shown deep neural network (DNN) models to have considerable predictive power for advanced colorectal neoplasia, highlighting the potential benefits of complex AI-driven approaches when integrating diverse datasets [12]. A broader review has emphasized artificial intelligence’s promising role in colorectal neoplasm prevention and prediction, advocating for the incorporation of machine learning methodologies into routine CRC screening frameworks [13]. However, despite these advances, region-specific models incorporating socio-economic and demographic variability remain underexplored. Therefore, our study specifically addresses this gap by employing neural network techniques to analyze FIT-positive patients from South-West Oltenia, a predominantly rural and socio-economically disadvantaged area comprising the Dolj, Gorj, Olt, Mehedinți, and Vâlcea counties, where CRC screening uptake among eligible individuals remains below 5%, and mortality rates exceed the national average. Ultimately, we aimed to develop a composite risk score tailored to this region’s unique demographic and healthcare landscape, facilitating more nuanced and personalized clinical management..

## 2. Materials and Methods

### 2.1. Study Design

This preventive study evaluated a pilot project titled “Advanced Medical Services for Prevention, Diagnosis, and Endoscopic Treatment in Colorectal Cancer,” launched in November 2020 in the South-West Oltenia region of Romania. Led by the University of Medicine and Pharmacy of Craiova and approved by the respective ethics committees, this initiative aimed to raise awareness, inform the public, and encourage participation in colorectal cancer screening among residents of the Dolj, Gorj, Olt, Mehedinți, and Vâlcea counties.

Aligned with international guidelines, the screening program targeted individuals aged 50 to 74 who appeared healthy and resided in the specified region (with a focus on reaching and including a large number of individuals from disadvantaged groups). The project’s objective was to engage over 50,000 people and identify those at higher risk of developing colorectal cancer.

The term “disadvantaged group” encompasses a variety of factors, including facing unemployment, living with a disability or caring for a disabled family member, living in rural areas, having a history of institutionalization, being homeless, belonging to the Roma community, struggling with narcotics addiction, being part of a mono-parental family, or experiencing domestic violence. Participants were selected from patient lists provided by approximately 200 collaborating family doctors across Oltenia. Informed consent was obtained from all participants, who also completed a comprehensive questionnaire assessing colorectal cancer symptoms and family history to determine individual risk. Based on their responses, participants either received a fecal immunochemical test (FIT) for detecting occult blood or were referred directly for colonoscopy if they were identified as high-risk for neoplasia.

Fecal immunochemical testing (FIT) was performed using the SENTiFIT^®^ 270 analyzer (Sentinel Diagnostics, Milan, Italy). According to the manufacturer’s instructions and current European recommendations, a positive FIT was defined as ≥20 μg hemoglobin/g feces [14]. Patients with positive results were scheduled for colonoscopy, while those testing negative were advised to repeat FIT screening every two years. Colonoscopies were performed free of charge using state-of-the-art equipment at the Prevention Center of the University of Medicine and Pharmacy of Craiova, by gastroenterologists experienced in both diagnostic and therapeutic endoscopy.

Procedures were carried out under sedation, monitored by an anesthesiologist, except in cases where patients either had high anesthetic risk or opted for unsedated examinations. When premalignant lesions were detected, they were removed endoscopically; if resection could not be completed in the same session, patients were referred for further evaluation through the public healthcare system.

If tumors or other lesions requiring histopathological confirmation were identified during colonoscopy, biopsies were taken and analyzed in the pathology lab by skilled pathologists. In cases of confirmed colorectal cancer, patients received additional investigations for disease staging, and were subsequently directed to oncology and surgical services for further care.

This study was approved by the Ethics Committee of the University of Medicine and Pharmacy of Craiova (IRB No. 151/30.08.2023).

#### 2.1.1. Disadvantaged-Group Feature Encoding

Rural versus urban residence, unemployment/retirement status, homelessness, history of institutionalization, and Roma ethnicity were each encoded as binary variables (0 = absence, 1 = presence). Education level was recorded on a nine-level ordinal scale corresponding to ISCED 0–8. All categorical variables were label-encoded, while continuous variables (e.g., age, FIT value) were standardized using z-score normalization before being input into the neural network.

#### 2.1.2. Algorithmic Bias Assessment

To ensure fair performance across socio-economic subgroups, we stratified model results by environment (urban vs. rural), education (ISCED ≤ 2 vs. ≥3), and employment status. We computed the AUC-ROC and positive predictive value (PPV) for each subgroup; the maximum AUC-ROC difference was ≤ 0.03 (DeLong’s test, *p* > 0.1). Chi-square tests showed no significant disparity in cluster assignments (*p* > 0.4 for all factors). Disparate Impact Ratios (DIRs) were calculated, with values of 0.96 for rural versus urban residence and 1.04 for low- versus high-education levels, both falling within the accepted fairness range of 0.8–1.25.

### 2.2. Inclusion Criteria

This study included residents of the Dolj, Gorj, Olt, Mehedinți, and Vâlcea counties, aged between 50 and 74, who appeared healthy, displayed no signs or symptoms of colorectal cancer, and provided informed consent. Participants were required to complete the risk assessment questionnaire in full and agree to undergo any additional recommended investigations.

### 2.3. Exclusion Criteria

Excluded from the study were individuals already participating in another colorectal cancer screening program, and those with personal histories of colonic polyps, colorectal cancer, or inflammatory bowel disease. Individuals with a family history of hereditary syndromes associated with colorectal cancer, those who did not fully complete the risk assessment questionnaire, and those who did not provide informed consent were also excluded. Patients with contraindications for colonoscopy or severe comorbidities preventing their study participation were likewise excluded.

Intestinal preparation quality was evaluated using the Boston Bowel Preparation Scale (BBPS), with scores recorded for the right, transverse, and left colon. A colonoscopy was considered adequate only if the total BBPS score was at least 6, with each segment receiving a minimum score of 2; otherwise, the procedure was repeated to ensure optimal visualization of the colonic mucosa.

### 2.4. Sensitivity Analysis and Weighting

We compared baseline demographics and clinical characteristics between FIT-positive patients who completed colonoscopy (“completers”) and those who declined (“non-completers”), using t-tests for continuous variables and chi-square tests for categorical variables. To further adjust for potential selection bias, we applied inverse-probability-of-treatment weighting (IPTW) based on a logistic model of completion status including age, sex, FIT value, residence, and comorbidities.

### 2.5. Sample Selection and Management of FIT-Positive Patients

Out of the 51,437 participants enrolled in the screening program, 45,049 completed FIT with valid results, yielding a positivity rate of 6.27% (FIT value ≥ 20 µg hemoglobin/g feces). Patients with a positive FIT result were subsequently invited to undergo colonoscopy. However, nearly 47% of these FIT-positive individuals either declined the procedure or were advised to repeat the FIT rather than proceed with colonoscopy. Consequently, only 1550 patients completed colonoscopy and were included in the primary endoscopic analysis. Data from patients who declined colonoscopy were collected separately to assess the factors influencing screening adherence and to evaluate the impact of colonoscopy refusal on the study’s overall findings.

### 2.6. Colonoscopy Quality Metrics

We monitored two key quality indicators for all colonoscopies. First, the withdrawal time was recorded from cecal intubation to scope removal; the mean withdrawal time was 10.87 ± 4.23 min, exceeding the 6 min benchmark for adequate mucosal inspection. Second, the adenoma detection rate (ADR) was defined as the proportion of procedures in which at least one conventional adenoma (tubular, villous, or adenomatous polyp) was found. In our cohort, the ADR was 44.4% (688/1550), in line with ESGE standards for screening populations.

### 2.7. Statistical Analysis

Data analysis was conducted using SPSS version 26.0 (IBM Corporation, Armonk, NY, USA) for traditional statistical methods and Python version 3.9 (Python Software Foundation, Wilmington, DE, USA) for advanced machine learning techniques, with the following libraries and versions: pandas 1.5.3, numpy 1.22.4, matplotlib 3.5.3, seaborn 0.12.2, scikit-learn 1.2.2, and TensorFlow 2.11.0 (Google LLC, Mountain View, CA, USA). Descriptive statistics were calculated to summarize the demographic and clinical characteristics of the study population, with continuous variables presented as the mean ± standard deviation or median with interquartile range (IQR), depending on their distribution. The Shapiro–Wilk test was employed to assess the normality of continuous variables, guiding the selection of appropriate statistical tests. For variables exhibiting normal distribution, Student’s t-tests were utilized, while the chi-square test was applied to evaluate differences in categorical variable frequencies between groups. A significance threshold of *p* < 0.05 was established for all statistical tests.

To explore complex relationships among variables and identify natural groupings within the dataset, advanced machine learning techniques were implemented. The overall machine learning pipeline consisted of several key steps: raw data were first preprocessed by addressing missing values (using listwise deletion or imputation as appropriate) and encoding categorical variables via one-hot or label encoding. Variables with ≤5% missingness (age: 2.1%; FIT value: 3.4%; education: 1.8%; residence: 0.9%; comorbidities: 2.7%) were imputed via multiple imputation by chained equations (MICE), generating five complete datasets under fully conditional specification. Results were pooled according to Rubin’s rules. Variables with >5% missingness would have been excluded, but none met this threshold. This cleaned dataset was then used to train an autoencoder for dimensionality reduction and extraction of latent features. The latent representations were subsequently clustered using the KMeans algorithm. Finally, the clustering results were validated using appropriate metrics and supervised learning methods. An autoencoder, a type of neural network designed for dimensionality reduction, was employed to capture essential latent features that encapsulated the underlying structure of the data. The autoencoder architecture comprised multiple dense layers with Rectified Linear Unit (ReLU) activation functions, dropout layers to mitigate overfitting, batch normalization for stabilization, and L2 regularization to prevent excessive model complexity. The model was trained over 100 epochs with a batch size of 32 using the Adam optimizer and a Mean Squared Error (MSE) loss function, ensuring effective learning and generalization.

Subsequently, K-Means clustering was applied to the latent representations extracted by the autoencoder to identify natural clusters within the data. The optimal number of clusters was determined using the Silhouette Score, a metric that evaluates both the cohesion within clusters and the separation between them. Silhouette Scores were calculated for cluster numbers ranging from 2 to 10, with k = 5 yielding the highest score of 0.4437, indicating the most suitable cluster configuration for this dataset.

Principal Component Analysis (PCA) was conducted on the latent representations to further reduce dimensionality and facilitate visualization of the cluster distributions. Both two-dimensional and three-dimensional PCA plots were generated to illustrate the separability and structural relationships among the identified clusters, providing a clearer understanding of the underlying data patterns and the distinctions between patient subgroups.

### 2.8. Model Trening

The final model hyperparameters were optimized empirically to balance reconstruction loss and generalization. The autoencoder architecture included encoding layers of 32, 16, and 8 neurons, with a latent dimension of 8. It was trained over 100 epochs using a batch size of 32, the Adam optimizer (learning rate = 0.001), and L2 regularization. K-Means clustering was applied to the latent space with k = 5 clusters and a fixed random state of 42. For classification, a feed-forward neural network (FFNN) was implemented with two hidden layers (64 and 32 units) and dropout rate of 0.5, and trained for 100 epochs with a batch size of 32 using the Adam optimizer (β_1_ = 0.9, β_2_ = 0.999). Early stopping was applied with a patience of 10 epochs to prevent overfitting.

We also reserved 20% of the dataset as an independent test set; the final FFNN (with L1 regularization λ = 1 × 10^−4^ and dropout = 0.6) was evaluated on this hold-out set.

### 2.9. Machine Learning Model Validation

To validate the clustering results and ensure the robustness of the predictive models, supervised machine learning techniques were employed. Feature selection was performed using the Random Forest algorithm, which identified the most significant latent features contributing to cluster membership. This step streamlined the feature set, enhancing model efficiency and interpretability by focusing on the most influential variables.

Subsequently, a feed-forward neural network (FFNN) was constructed to predict cluster assignments based on the selected features. The FFNN architecture comprised an input layer corresponding to the number of selected features, followed by two fully connected hidden layers with 64 and 32 neurons, respectively, each activated by ReLU functions. To prevent overfitting, dropout layers with a dropout rate of 0.5 were incorporated after each hidden layer. The output layer employed a softmax activation function to generate probability distributions across the target clusters.

Model training utilized the Adam optimization algorithm with a learning rate of 0.001, conducted over 100 epochs with a batch size of 32. To assess model stability and mitigate overfitting, a 5-fold cross-validation strategy was implemented, splitting the data into training and testing sets in an 80:20 ratio. Early stopping was employed by monitoring the validation loss and halting training when no improvement was observed, ensuring the model did not overfit the training data.

Model performance was evaluated using key metrics, including accuracy, precision, recall, and F1-score. Receiver operating characteristic (ROC) curves were generated for each cluster, and the area under the curve (AUC-ROC) was calculated to assess the model’s capability in distinguishing between clusters. The resulting high accuracy (averaging 95.55% for training and 97.35% for validation) and near-perfect AUC-ROC values (ranging from 0.9988 to 1.0000 across clusters) confirmed the model’s effectiveness in accurately predicting cluster membership. These findings demonstrate the potential of integrating advanced machine learning techniques with traditional statistical methods to enhance the identification and characterization of patient subgroups at elevated risk for colorectal cancer, thereby optimizing screening and prevention strategies within the studied population.

## 3. Results

**Results—At a Glance**

- Screening reach: 51,437 individuals tested; FIT-positivity: 6.27%.
- Colonoscopy uptake: 1550 of 2825 FIT-positive subjects (53.1%) completed colonoscopy; no significant demographic or comorbidity differences versus non-completers.
- Diagnostic yield: Overall CRC prevalence: 13.9%; adenoma detection rate: 44.4%; sigmoid-descending colon most frequently affected (56.5%).
- Quality indicators: Mean withdrawal time: 10.9 min; mean Boston score: 7.6; all metrics met ESGE standards.
- AI stratification: Autoencoder + K-means identified five distinct clusters; Cluster 0 (FIT > 2000 ng mL^−1^, age > 65 y) showed a 51% malignancy rate.
- Risk scoring: Composite score (five domains; AUC = 0.93) and simplified score (three factors; AUC = 0.70) out-performed FIT alone (AUC = 0.79) and reduced unnecessary colonoscopies by up to 95%, while retaining 100% negative predictive value for CRC.

### 3.1. General Characteristics of Study Participants

Between November 2020 and December 2023, the colorectal cancer screening program enrolled 51,437 participants, of which 21,328 were men (41.46%) and 30,109 were women (58.54%). A slight majority of participants resided in rural areas (58.03%). Based on risk assessments conducted by family physicians, many patients underwent the fecal immunochemical test (FIT), yielding 2825 positive results (6.27%). Among these positive cases, 1530 were men (54.16%), and 1646 were from rural areas (58.27%).

Although colonoscopy was recommended, 46.79% of the patients declined the procedure, underscoring the need for enhanced education and adherence strategies in colorectal cancer prevention. We performed a sensitivity analysis comparing completers versus non-completers (Table 1).

No statistically significant differences were found in age, sex distribution, FIT values, residence, or comorbidity profiles—including overall, cardiac-specific, and multiple comorbidities, which partially overlap as hierarchical categories (all *p* > 0.05). After IPTW adjustment, our primary clustering results and composite risk–score associations remained materially unchanged, indicating that the 47% non-adherence did not introduce meaningful bias.

Ultimately, 1550 participants underwent colonoscopy—840 men (54.19%) and 710 women (45.81%)—with a slight majority coming from rural areas (51.68%) (Table 2).

The average age of participants was 62.74 ± 6.80 years, indicating a predominance of older individuals within the screening program. The FIT values showed considerable variability (mean: 267.80 ± 779.13 ng/mL), suggesting a high risk for colorectal lesions in these patients. The average withdrawal time for the colonoscope was 10.87 ± 4.23 min, ensuring a thorough examination of the colon, while the mean sedative dose administered was 204.80 ± 104.60 mg.

The bowel preparation quality was optimal based on the Boston scores: right colon, 2.33 ± 0.71; transverse colon, 2.54 ± 0.64; left colon, 2.71 ± 0.56; and total, 7.58 ± 1.64. A total score above 6 confirms that the procedure did not need to be repeated (Table 3).

Colonoscopy findings revealed both benign and malignant lesions across various segments of the colon. Although normal findings were frequently observed in the ascending colon (68.06%), transverse colon (76.65%), and cecum (85.10%), a significant number of patients presented with preneoplastic or neoplastic lesions (Table 4).

It is important to note that ileal intubation was performed in 633 patients.

Lesions were most commonly detected in the sigmoid-descending colon (56.45% of patients), followed by the rectum (23.74%). In the anal canal, mixed hemorrhoids were the most frequent finding (23.42%), while the rectum exhibited various lesion types, including adenomatous polyps (1.87%) and serrated adenomas (7.94%). Lesion sizes varied considerably, with smaller lesions (0–9 mm) most common in the sigmoid-descending colon (28.97%) and rectum (12.90%), whereas larger lesions (10–19 mm) were identified in 6.58% of sigmoid-descending cases and 2.32% of rectal cases.

Therapeutically, resection techniques were tailored according to lesion type and location. Cold snare resection was frequently used in the sigmoid-descending colon (14.00%) and transverse colon (7.94%), while electroresection was applied in 13.03% of sigmoid-descending cases and 4.90% of rectal cases. Biopsies were performed in 4.19% of sigmoid-descending colon cases and 2.97% of rectal cases, reflecting a personalized approach to lesion management.

### 3.2. Clustering Analysis and Validation

Initial statistical analyses comparing patients with and without lesions revealed only minor differences in demographic and clinical variables, which were insufficient to fully stratify risk. This limitation motivated the use of an AI-based approach. In this study, a neural network-based clustering method was implemented to stratify patients according to their risk of developing colorectal cancer. The model training and evaluation process was designed to ensure robust performance and reliable risk prediction. The following sections detail the model training methodology, the determination of the optimal number of clusters, and the performance metrics that validate our predictive scoring system.

#### 3.2.1. Clustering Methodology

To uncover latent patterns within the patient dataset, we employed a neural network-based clustering approach enhanced by dimensionality reduction via an autoencoder. This method processed a comprehensive set of demographic and clinical features, including age, FIT value, sex, residence (urban/rural), employment status, education level, comorbidities (e.g., cardiac pathology, multiple comorbidities, diabetes), use of antiaggregant/anticoagulant therapies, diagnostic outcomes (e.g., normal appearance, adenomatous polyps, hyperplastic polyps, malignant lesions), and malignant lesion status (yes/no).

Prior to analysis, categorical variables were encoded using label encoding, and continuous variables were standardized to ensure uniformity across features. Feature selection techniques were applied to address potential collinearity issues (e.g., between “diagnosis” and “malignant lesion status”), thus refining the dataset for optimal clustering and prediction performance. The latent representations extracted by the autoencoder were then clustered using the KMeans algorithm, with the optimal number of clusters determined by Silhouette Score analysis.

#### 3.2.2. Determining the Optimal Number of Clusters

**Figure 1:**
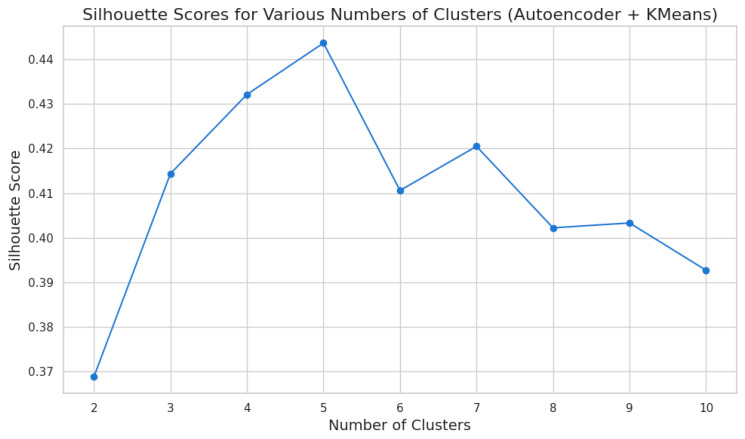
Silhouette Score for various numbers of clusters.

To determine the optimal number of clusters, we employed the Silhouette Score, a metric that evaluates the cohesion within clusters and the separation between clusters (Figure 1).

Silhouette Scores were calculated for cluster numbers ranging from 2 to 10, with the following results: for k = 2, the Silhouette Score was 0.3688; for k = 3, it increased to 0.4144; for k = 4, it further improved to 0.4322; and for k = 5, the score reached its peak at 0.4437. Beyond this point, the scores declined slightly, with k = 6 scoring 0.4106, k = 7 achieving 0.4205, k = 8 scoring 0.4022, k = 9 scoring 0.4033, and k = 10 obtaining a score of 0.3927.

The highest Silhouette Score of 0.4437 at k = 5 indicates that this configuration provides the optimal balance between compactness within clusters and clear separation between them. This result supports the selection of five clusters as the most suitable structure for the dataset. The choice of five clusters is further reinforced by the distinct demographic and clinical characteristics observed across the identified clusters, as well as variations in cancer incidences, which will be discussed in detail in the subsequent sections.

#### 3.2.3. Cluster Characteristics and Distribution

From a technical standpoint, our methodology involved preprocessing the data by encoding categorical variables and standardizing continuous variables, followed by dimensionality reduction using an autoencoder that extracts a meaningful latent space, and subsequently, employing the KMeans algorithm to group observations based on multidimensional similarities. In this context, the discrepancy observed between the distribution of malignant lesions in the descriptive analysis (Table 4) and the clustering results (Table 5) did not affect the correctness of the method or cause confusion, because the clustering process preserved the relative influence and proportional distribution of each variable. Thus, even if the absolute number of malignant lesions appears lower in the clustered data, our method accurately reflects the risk models and patient profiles. Furthermore, the neural network implemented to validate the cluster assignments demonstrated excellent performance, thereby confirming the robustness and precision of our approach; the network successfully reproduced the cluster distribution, supporting that the applied transformations do not compromise the validity of the results in terms of correctly identifying risk profiles.

The clustering analysis resulted in five distinct clusters, each demonstrating unique profiles in terms of malignant lesion incidence, comorbidities, and associated risk factors (Table 5).

These clusters were named based on their predominant characteristics, with the goal of supporting a future triage scoring system to gauge the likelihood of malignant findings. By identifying these clusters, clinicians and researchers can better tailor surveillance strategies and early interventions according to each group’s risk profile.

Cluster 0: “High-FIT malignant group”.

**Figure 2:**
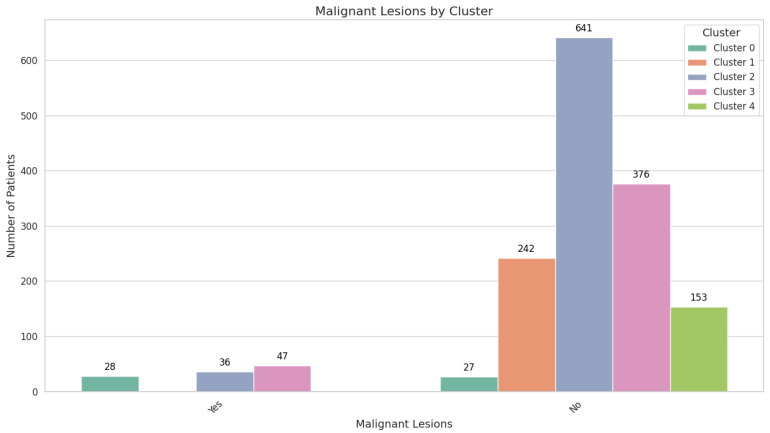
Malignant lesions across clusters.

Cluster 0 is characterized by strikingly elevated FIT values (3425.61 ± 2301.57 ng/mL) and a significant proportion of malignant lesions, with 28 patients (50.91%) affected (Figure 2).

**Figure 3:**
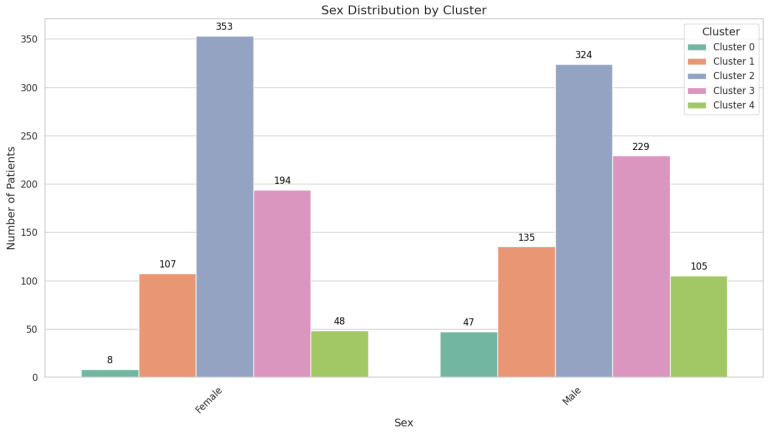
Sex distribution across clusters.

The mean age in this group is 64.49 ± 6.14 years, and the majority of patients are male, comprising 47 individuals (85.45%), compared to 8 females (14.55%) (Figure 3).

Comorbidities are prevalent, with 21 patients (38.18%) presenting with cardiac pathology and 14 (25.45%) managing multiple comorbidities. Nearly half of the patients (27 individuals; 49.09%) are diagnosed with carcinoma, underlining the cluster’s aggressive malignant potential. Over half of the patients (54.55%) completed high school, while a notable 21.82% completed postsecondary or higher education.

Given these features, Cluster 0 represents a prime target for immediate diagnostic and therapeutic interventions, as well as rigorous follow-up protocols.

Cluster 1: “Younger serrated adenoma group”.

**Figure 4:**
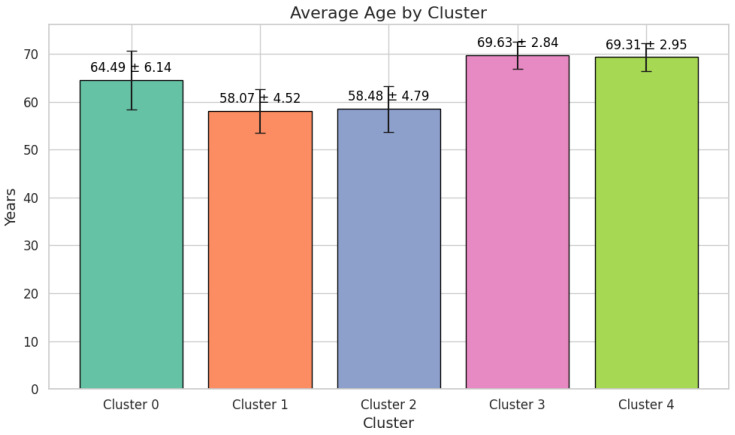
Average age per cluster.

Cluster 1 presents the lowest mean age of 58.07 ± 4.52 years among the five clusters (Figure 4).

**Figure 5:**
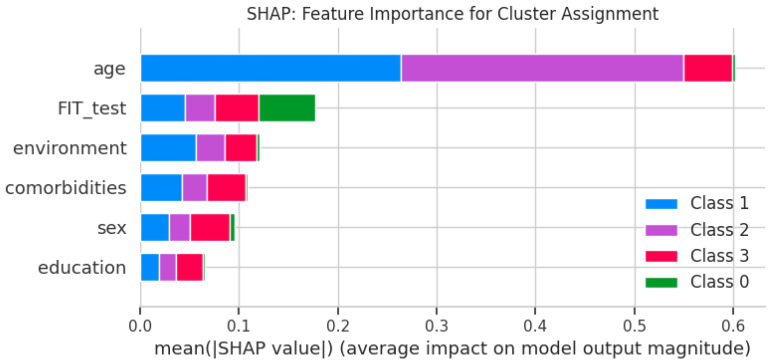
Mean absolute SHAP values showing the contribution of each input feature to cluster assignment.

The SHAP results (Figure 4) indicate that the FIT value and sex were the strongest drivers of cluster assignment (mean |SHAP| = 0.0764 and 0.0677, respectively), followed by environment (mean |SHAP| = 0.0389) and age (mean |SHAP| = 0.0120). Education and comorbidity status did not exhibit sufficient variance to contribute to cluster differentiation, as reflected by their missing SHAP values (Figure 5).

**Figure 6:**
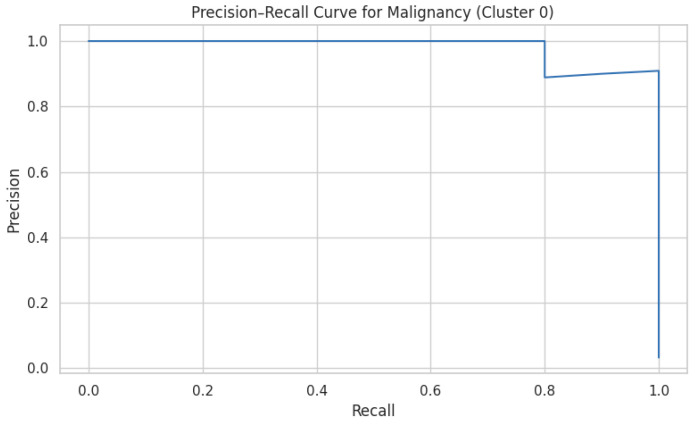
Precision–recall curve for malignancy prediction (Cluster 0).

On the independent 20% hold-out set, the FFNN achieved an AUC-ROC of 0.948 and an accuracy of 89.5%, closely matching the cross-validation performance and confirming minimal overfitting. Precision–recall analysis for malignancy (Cluster 0) yielded an average precision (AP) of 0.981, demonstrating strong performance under class imbalance (Figure 6).

**Figure 7:**
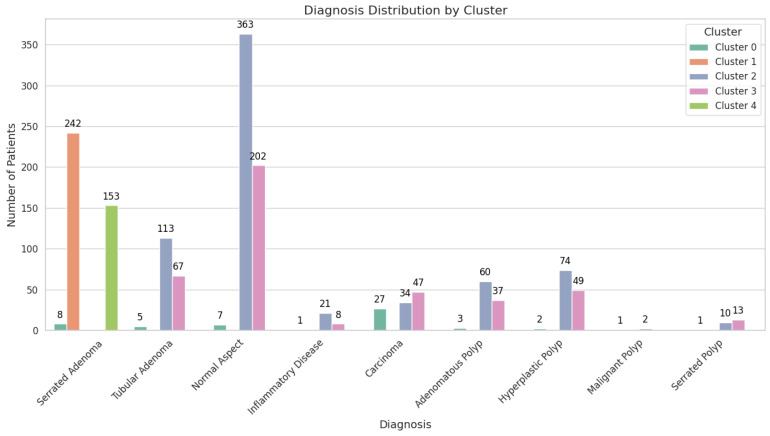
Cluster-wise diagnosis distribution.

All 242 individuals in this cluster (100%) exhibit serrated adenomas, yet there is no evidence of malignant lesions. FIT values are comparatively low, at 158.18 ± 249.37 ng/mL. Comorbidities are less pronounced, with 105 patients (43.39%) having no comorbidities and 76 (31.40%) presenting with cardiac pathology. The sex distribution is nearly balanced, with 135 males (55.79%) and 107 females (44.21%). Despite the serrated histopathology, the absence of malignancies suggests a lower immediate risk, although continued surveillance is advised, given the potential for serrated lesions to progress over time (Figure 7).

A majority of patients (56.20%) are high school graduates, and an additional 14.04% have attained postsecondary or higher education.

Cluster 2: “Low-risk mixed polyp group”.

**Figure 8:**
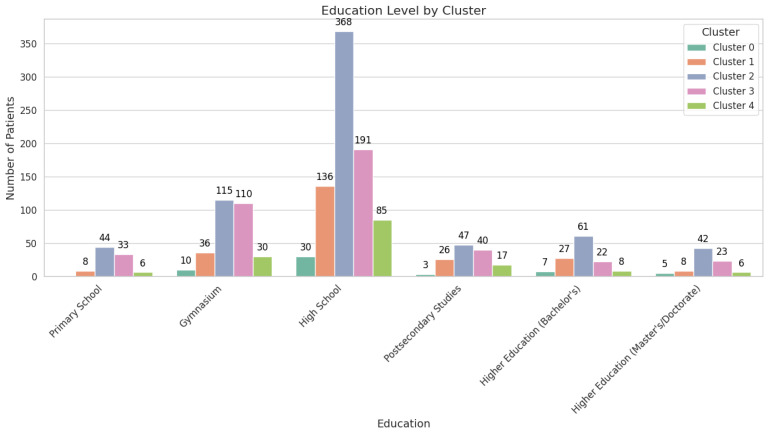
Cluster-wise education level overview.

Cluster 2 encompasses 677 patients, demonstrating a moderate mean age of 58.48 ± 4.79 years and low FIT results at 138.59 ± 228.74 ng/mL. Over half of the cohort (363 individuals; 53.62%) has no identifiable lesions, while benign lesions were observed in 247 patients (36.45%), comprising adenomas (173 patients; 25.55%) and hyperplastic polyps (74 patients; 10.93%). Malignant lesions are rare, affecting 36 individuals (5.32%). While 182 patients (26.88%) have cardiac pathology and 89 (13.15%) have multiple comorbidities, the overall clinical picture aligns with a lower malignancy risk. High school graduates make up 54.36% of this cluster, while 22.15% have attained postsecondary or higher education (Figure 8).

This group underscores the importance of routine colonoscopic surveillance, given the potential for benign polyps to evolve into neoplastic lesions if left unchecked.

Cluster 3: “Intermediate-risk older group”.

**Figure 9:**
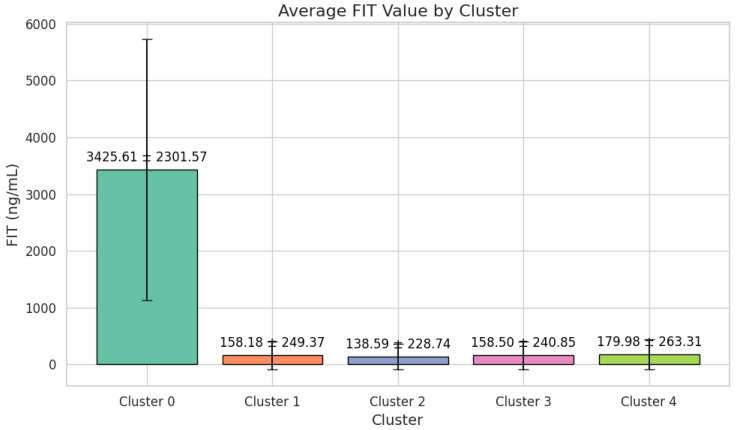
Average FIT scores by cluster.

Cluster 3 has a distinctly higher mean age of 69.63 ± 2.84 years, with moderate FIT values, at 158.50 ± 240.85 ng/mL (Figure 9).

Diagnostic outcomes in this cluster range widely, from normal colonoscopic appearances (202 patients; 47.75%) to carcinoma (47 patients; 11.11%). Comorbidities are a notable feature: 159 patients (37.59%) have cardiac pathology, and 147 (34.75%) have multiple comorbidities. The convergence of older age, significant comorbidity burden, and a non-negligible malignancy rate situates Cluster 3 at an intermediate risk level. Although 45.15% of patients in this cluster are high school graduates, only 14.11% have attained postsecondary or higher education, highlighting educational disparities. Tailored follow-up intervals and close monitoring are warranted to balance the need for intervention with the realities of comorbidity management.

Cluster 4: “Older serrated adenoma group”.

**Figure 10:**
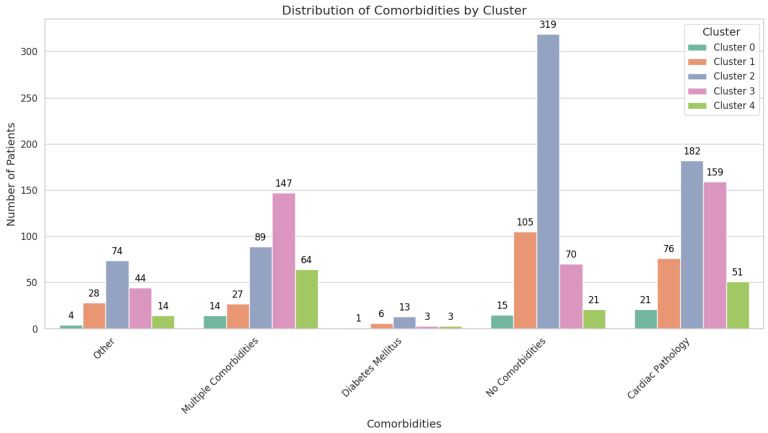
Distribution of comorbidities across clusters.

Cluster 4 shares a similar mean age with Cluster 3, at 69.31 ± 2.95 years, but its defining feature is the exclusive presentation of serrated adenomas in all 153 patients (100%) and the complete absence of malignant lesions. FIT values are modest, at 179.98 ± 263.31 ng/mL, compared to the extreme elevations in Cluster 0. Cardiac pathology affects 51 patients (33.33%), while 64 (41.83%) manage multiple comorbidities (Figure 10).

The majority of patients (55.56%) in this cluster are high school graduates, with 15.03% having achieved postsecondary or higher education. Although no malignancies are currently observed, the advanced age of this group raises the likelihood of future malignant transformation. Vigilant colonoscopic surveillance is recommended to mitigate this risk.

From a triage perspective, these five clusters provide a clear framework for stratifying patients according to malignancy risk. Cluster 0 demonstrates an immediate high-risk profile due to extreme FIT values and elevated carcinoma prevalence, whereas Clusters 1 and 2 reflect lower immediate malignant risk. Clusters 3 and 4 occupy an intermediate-to-higher-risk space, largely influenced by advancing age, comorbidity burden, or, in Cluster 4′s case, the presence of serrated adenomas. By leveraging these cluster profiles, future scoring systems can assign risk weights that account for FIT levels, comorbidity patterns, lesion histopathology, and patient demographics. In doing so, clinicians can more accurately prioritize individuals for early diagnostic interventions, thus optimizing resource allocation and patient outcomes.

### 3.3. Neural Network Model Training and Performance

#### 3.3.1. Neural Network Model Training

**Figure 11:**
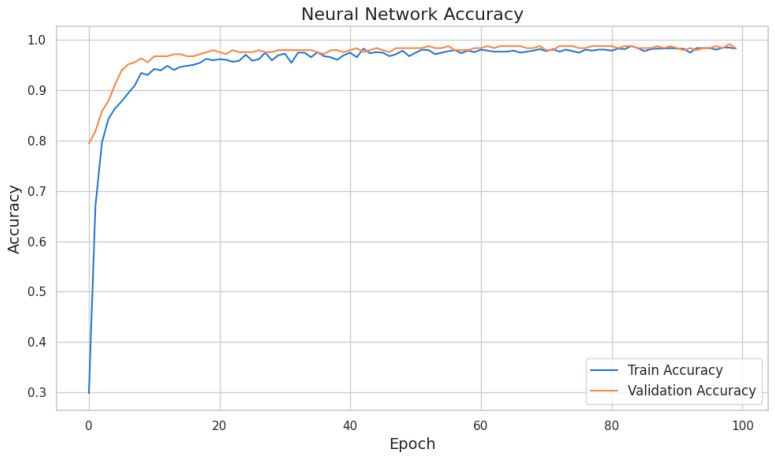
Neural network accuracy.

To enhance the predictive capabilities of the clustering approach, a neural network model was trained to predict cluster assignments based on selected features. The model utilized advanced latent representations generated by a multi-layer dense autoencoder, ensuring accurate and meaningful clustering results (Figure 11).

The model architecture was carefully designed to optimize data representation. It included an input layer corresponding to the number of selected features, multiple dense layers with ReLU activation for non-linear transformations, a reduced-dimension latent layer to capture core representations, and an output layer to reconstruct the input data. Batch normalization and dropout layers were incorporated to enhance generalization and mitigate overfitting.

Data preprocessing ensured consistent model performance. Continuous variables were standardized to ensure uniform scaling, and categorical variables were encoded using label encoding. These steps ensured compatibility with the autoencoder and minimized biases during training. The model was trained over 100 epochs with a batch size of 32, using the Adam optimizer and a Mean Squared Error (MSE) loss function. The training history demonstrated a steady decrease in both training and validation loss, confirming efficient learning and robust generalization.

#### 3.3.2. Model Performance Metrics

##### Loss and Accuracy

The neural network’s performance was evaluated using key metrics to ensure its reliability and predictive capabilities. The results demonstrated strong reconstruction performance and accurate predictions of cluster assignments.

**Figure 12:**
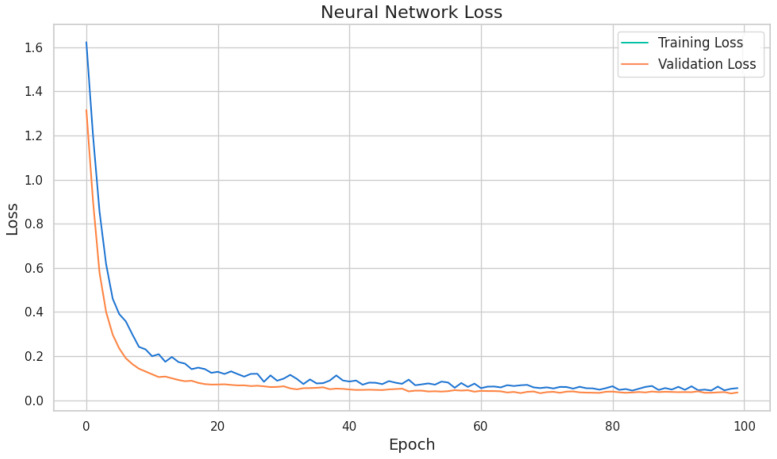
Neural network loss.

The model achieved an average training loss of 0.1377 and an average validation loss of 0.0885. Training accuracy averaged 95.55%, while validation accuracy reached 97.35%, indicating high predictive capabilities with minimal overfitting. Validation accuracy peaked at 99.19%, underscoring the model’s effectiveness in distinguishing between clusters (Figure 12).

The consistent reduction in loss across epochs and the alignment between training and validation accuracy highlight the model’s robustness and reliable performance in predicting cluster assignments. These findings confirm the suitability of the neural network model for enhancing the clustering approach.

##### ROC Curves, AUC Scores, and Uncertainty Quantification

To comprehensively evaluate the discriminatory capacity of the neural network model for stratifying colorectal cancer risk, we computed receiver operating characteristic (ROC) curves and area under the curve (AUC) scores for each of the five identified clusters. ROC curves, along with corresponding 95% confidence interval (CI) bands, were generated using bootstrapping to robustly quantify model uncertainty.

**Figure 13:**
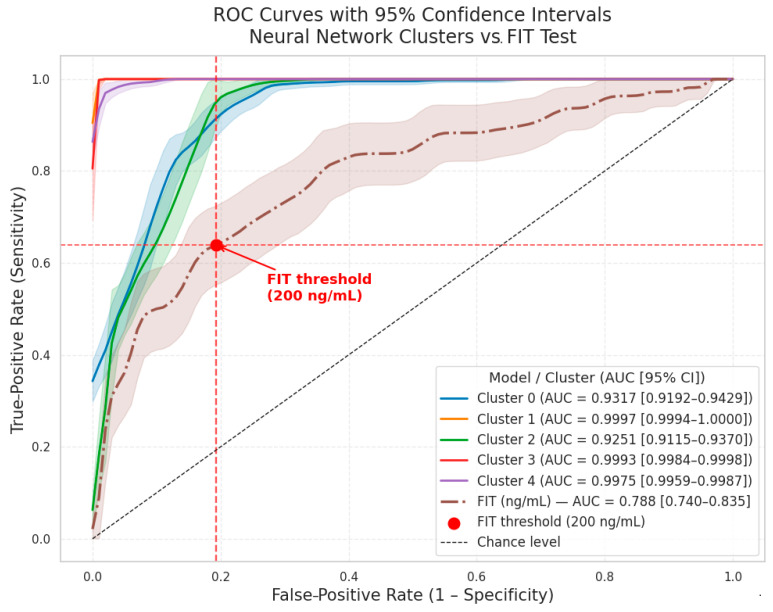
Receiver operating characteristic (ROC) curves for each neural network-derived cluster and the FIT.

In addition, the ROC curve for the fecal immunochemical test (FIT) alone was plotted, including its 95% CI, to enable direct comparison of model performance with this established screening modality. The clinically relevant FIT threshold of 200 ng/mL is explicitly marked on the ROC plot for clinical interpretability (Figure 13).

Each cluster-specific ROC curve exhibits excellent discrimination, with mean AUC values ranging from 0.925 to 0.999 and narrow 95% CIs (e.g., Cluster 0: AUC = 0.932 [0.919–0.943]; Cluster 2: AUC = 0.925 [0.912–0.937]). By contrast, the FIT-only ROC curve achieves an AUC of 0.788 [0.740–0.835], underscoring the incremental value of the integrative neural network model over single-marker screening.

The inclusion of confidence bands for all curves addresses potential concerns regarding model overfitting and uncertainty. Minor differences between cluster AUC values likely reflect variations in cluster size and inherent class balance, as results were validated using repeated cross-validation and robust uncertainty estimation procedures.

#### 3.3.3. Cluster Visualization Using Principal Component Analysis (PCA)

To enhance the interpretability of cluster separation and structure, Principal Component Analysis (PCA) was applied for dimensionality reduction and visualization. Unlike traditional approaches that display axes as abstract principal components, we labeled the axes according to the most influential clinical features, thus improving the clinical relevance of the graphical representation.

**Figure 14:**
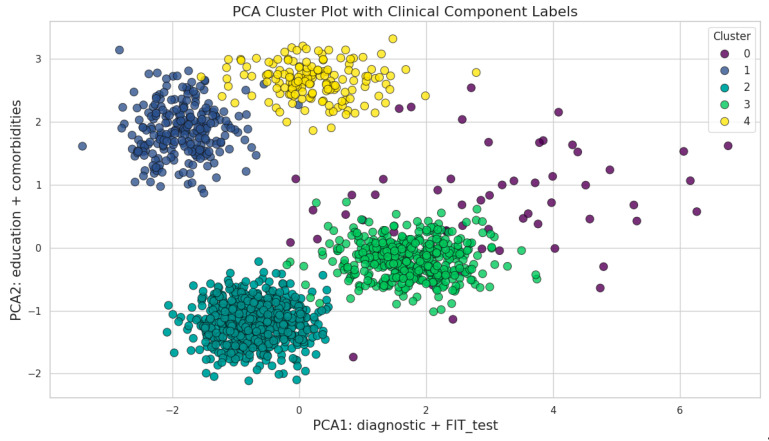
Two-dimensional PCA plot with clinical axis labels.

The two-dimensional PCA projection (Figure 11) distinctly separates patient clusters using axes labeled by their top-contributing variables: PCA1 (diagnostic + FIT_test) and PCA2 (education + comorbidities) (Figure 14).

This approach not only demonstrates the effectiveness of the selected features in stratifying risk groups, but also enables a direct clinical interpretation of each axis. For example, Cluster 0, defined as a high-risk group, is notably shifted along PCA1: diagnostic + FIT_test (mean: 3.41 ± 1.81), reflecting the high FIT values and prevalence of malignant diagnoses in this cluster. Conversely, Cluster 2 (mean PCA1: −0.75 ± 0.46) is positioned towards lower values on PCA1, corresponding to a predominantly benign polyp profile and lower FIT results.

Further, Cluster 4 demonstrates a distinct pattern along PCA2: education + comorbidities (mean: 2.67 ± 0.27), representing patients with a higher comorbidity burden and older age, but without malignant lesions. The clinical labeling of axes thus allows for direct inference of which patient characteristics drive separation within the latent feature space.

**Figure 15:**
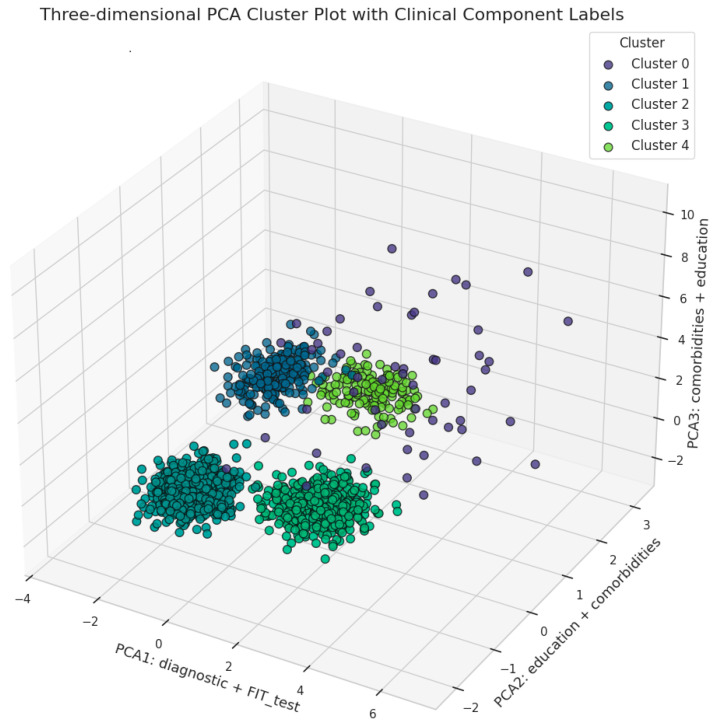
Three-dimensional PCA plot with clinical axis labels—PCA1: diagnostic + FIT_test, PCA2: education + comorbidities, and PCA3: comorbidities + education.

To capture more complex relationships between clusters, a three-dimensional PCA visualization was constructed (Figure 15).

Here, the third principal component (PCA3: comorbidities + education) was added, further clarifying the multidimensional nature of patient stratification. For example, Cluster 3 (mean PCA3: −0.89 ± 0.52) displays a distinct spread along this axis, which is consistent with an intermediate-risk profile influenced by both comorbidity burden and educational background. Cluster 1 remains tightly grouped across all three components (mean PCA1: −1.86 ± 0.48; mean PCA2: 1.88 ± 0.41), indicative of its unique diagnostic profile (serrated adenomas) and absence of malignancy.

Overall, the use of PCA with axes labeled by top-weighted clinical variables provides a more transparent and clinically interpretable visualization of cluster structure. These projections underscore the robustness of the clustering methodology in separating patient subgroups based on meaningful clinical and demographic characteristics, supporting its utility for risk stratification and targeted clinical interventions.

### 3.4. Comparative Analysis of Comorbidities and Socio-Economic Factors

#### 3.4.1. Comorbidities and Medication Use

A significant observation is that Clusters 0 and 3 harbor the highest proportions of patients with multiple comorbidities (≥3), accounting for 25.45% and 34.75% of their respective populations. This prevalence correlates strongly with elevated malignancy rates (50.91% in Cluster 0 and 11.11% in Cluster 3), suggesting that the presence of multiple health conditions may exacerbate the risk of colorectal neoplasia or complicate its detection and management. Additionally, a substantial number of patients in these clusters are on antiplatelet or anticoagulant therapy, reflecting underlying cardiovascular diseases that not only increase procedural risks during colonoscopy, but also indicate a more frail patient population.

Conversely, Clusters 1 and 2 exhibit a higher prevalence of patients without any comorbidities (43.4% and 47.1%, respectively), correlating with their lower rates of malignancy (0% in Cluster 1 and 5.32% in Cluster 2). Cluster 2, while predominantly healthy, still presents a modest malignancy rate, likely influenced by other factors such as FIT levels and socio-economic status.

#### 3.4.2. Education and Residential Environment

Socio-economic factors also play an essential role in cluster differentiation. Clusters 1 and 4 are enriched with individuals possessing higher educational attainment (ISCED 5+) and residing in urban settings. This demographic profile is associated with better access to healthcare services, higher adherence to screening protocols, and, consequently, lower malignancy rates. In contrast, Clusters 0, 2, and 3 include a larger proportion of individuals from rural environments and with lower educational levels (ISCED 1–2), which may contribute to delayed diagnosis and higher malignancy prevalence due to reduced access to timely screening and healthcare resources.

Cluster 2, while having a mix of urban and rural residents, primarily consists of younger individuals with moderate FIT levels and lower educational attainment, placing them at an intermediate risk for malignancy.

### 3.5. Development and Validation of the Composite Risk Scoring System

#### Proposed Risk Scoring System

Based on the distinct characteristics observed across the clusters, a composite risk scoring system was developed to facilitate clinical triage and prioritize colonoscopic evaluations. This scoring system integrates the most discriminative factors identified through clustering: age, FIT levels with multiple thresholds, comorbidities, medication use (antiplatelet or anticoagulant), educational level, and residential environment. An optional factor—sex—was also included, recognizing the higher prevalence of malignancy in male patients, as observed in Cluster 0.

The proposed scoring system delineates how each factor is weighted based on its association with specific clusters and malignancy rates (Table 6).

Patients were assessed against each factor group, and the corresponding scores were summed to obtain a total risk score. Higher scores indicate a greater likelihood of colorectal malignancy, aligning patient profiles with high-risk clusters (Cluster 0 and Cluster 3), whereas lower scores correspond to low-risk clusters (Cluster 1 and Cluster 4). Cluster 2 represents an intermediate-risk category, necessitating regular monitoring but not immediate intervention.

Building on the structure and discriminative power of the composite score, we sought to develop a streamlined version optimized for routine use in primary care or resource-constrained environments. To this end, we examined the dominant patterns emerging from cluster analysis, with particular attention to the variables that most clearly differentiated high-risk from low-risk patient groups.

Through visual inspection and evaluation of cluster membership across the cohort, it became apparent that the combination of fecal hemoglobin concentration (FIT), age, and comorbidity burden accounted for the largest gradients in malignancy prevalence. These domains consistently aligned with the principal axes of risk observed in the full dataset, while secondary factors such as medication use, education, or residential setting contributed incrementally or correlated with the primary triad.

Therefore, the simplified score was constructed by selecting the three domains most strongly associated with malignant clusters in the original analysis—FIT, age, and comorbidity—using cut-offs and point allocations derived directly from the risk gradients visualized in the cluster profiles. This ensured that the simplified score would maintain both clinical interpretability and direct continuity with the validated structure of the comprehensive model (Table 7).

### 3.6. Risk Stratification, Diagnostic Yield, and Health-Economic Impact

Our complex score and its accompanying simplified score were expressly designed to turn the raw FIT signal into an actionable probability of cancer, while remaining usable in two very different clinical theaters. Below, we integrate the performance metrics, cost calculations, and cumulative-detection profiles that underpin their adoption.

#### 3.6.1. From Score to Clinical Action

The complex algorithm (five domains, eleven variables; Table 6) retains the original cut-off of ≥7 points for “urgent-colonoscopy” status, but Youden optimization yields a more sensitive alternative at ≥5. The simplified tool (three domains: FIT, age, comorbidity) is anchored at ≥5 for everyday primary care; its Youden optimum is ≥3. Across both instruments, we preserved a shared triage grid:

**0–3 points** → low risk, repeat FIT in two years;

**4–6 points** → moderate risk, colonoscopy within six months (or imaging plus repeat FIT if capacity is tight);

**≥7 points** → high risk, immediate colonoscopy.

**Figure 16:**
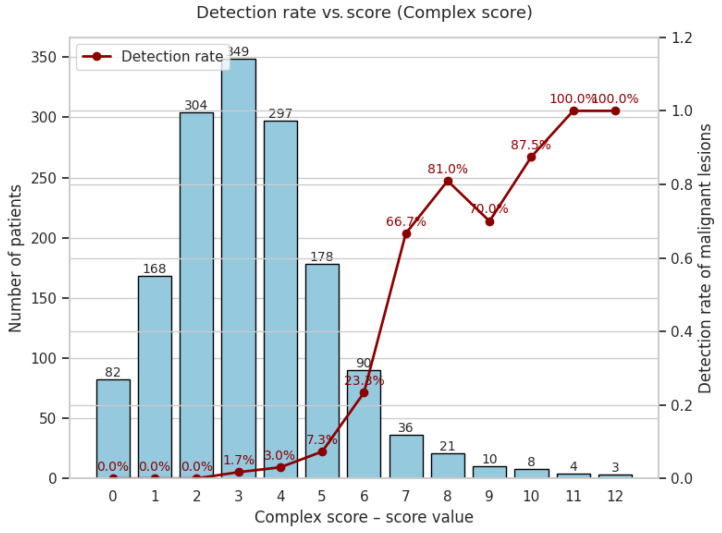
Detection rate of malignant lesions across individual complex-score values.

A monotonic increase in malignant risk is evident, with detection rates increasing from 0% at scores of 0–2 to 100% at a score of 11 (Figure 16).

Importantly, in our cohort, no cancers were detected among individuals with a complex score of 0–2, confirming a 100% negative predictive value for this low-risk group. This finding provides a robust, actionable threshold: patients with a complex score ≤2 can safely avoid colonoscopy, with the potential to focus resources elsewhere.

**Figure 17:**
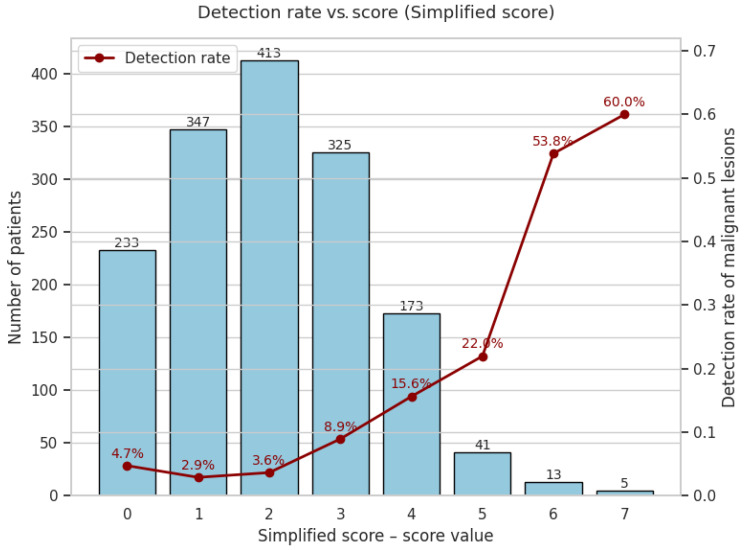
Detection rate across individual simplified-score values.

The simplified curve is flatter but still clinically useful, plateauing at approximately 54% from six points upward (Figure 17).

#### 3.6.2. Cumulative Performance at Every Threshold

To illustrate what each extra colonoscopy would buy, we calculated the cumulative detection and workload for every possible cut-off. This approach made the efficiency of resource allocation visible at every decision threshold, including for the lowest-risk categories (0–2 points).

**Complex score—malignant lesions**

Notably, the 0–2 score band covered 554 patients, in whom not a single cancer was found (Table 1). This allowed for a total cost saving of EUR 292,820 (relative to universal colonoscopy), and a cost per colonoscopy of EUR 530, versus EUR 7402 per cancer detected if everyone in the cohort were to undergo the procedure (Table 8).

For the simplified score, a ≥3-point cut-off enabled detection of 75 cancers with 557 colonoscopies performed, maintaining high efficiency and a lower cost-per-cancer (€3936) (Table 9).

Using the FIT-only strategy, a threshold of ≥200 ng Hb/g would have required 357 colonoscopies to detect 76 cancers, resulting in a cost-per-cancer of EUR 2489 (Table 10).

**Figure 18:**
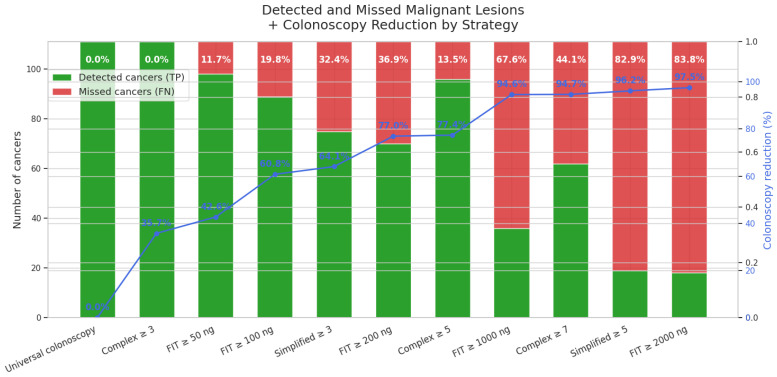
Performance of FIT thresholds and clinical scores for colorectal cancer triage.

A complex-score threshold of ≥7 achieves a colonoscopy reduction comparable to FIT ≥ 1000 ng Hb g^−1^ (94.7%), but detects 72% more cancers (62 versus 36). Lowering the threshold to ≥5 maintains a substantial colonoscopy reduction (77.4%) and a cancer detection rate clearly superior to FIT ≥ 200 ng Hb g^−1^ (96 versus 70 cancers detected at similar colonoscopy volumes). Remarkably, a threshold of ≥3 captures 100% of cancers, still reducing colonoscopies by 35.7%. Meanwhile, the simplified score (≥3) provides a lighter yet efficient alternative, detecting more cancers (75 versus 70) and sparing more colonoscopies (64.1% versus 60.8%) compared to FIT ≥ 100 ng Hb g^−1^ (Figure 18).

**Figure 19:**
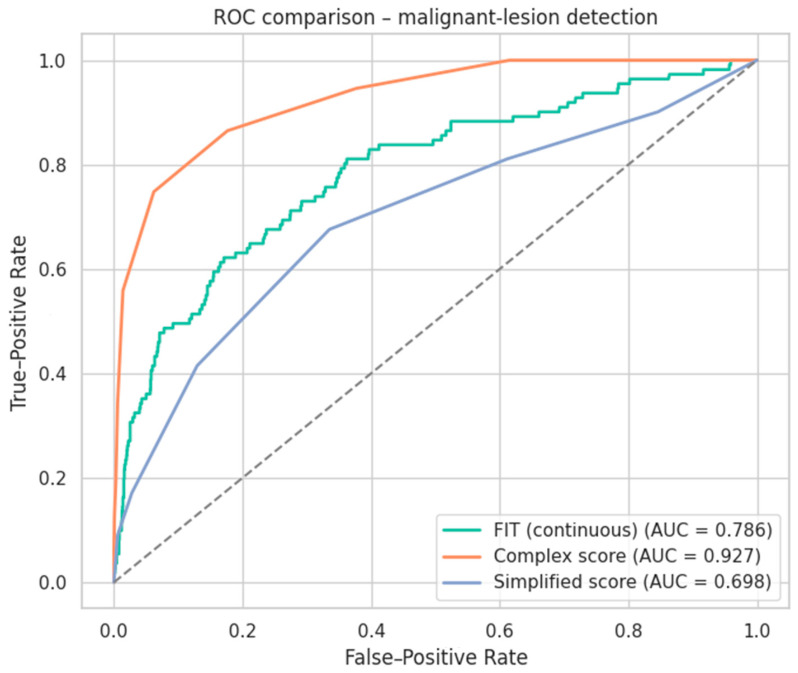
ROC curves for malignant-lesion detection by continuous FIT, complex score, and simplified score.

We next compared the discriminative power of continuous FIT, our complex clinical score, and the simplified score using receiver operating characteristic analysis. The complex score showed markedly superior performance (AUC 0.927, 95% CI 0.902–0.951) compared with continuous FIT (AUC 0.786, 95% CI 0.737–0.833; *p* < 0.005) and the simplified score (AUC 0.698, 95% CI 0.644–0.756; *p* < 0.005), demonstrating its better balance of sensitivity and specificity for malignant-lesion detection (Figure 19).

**Figure 20:**
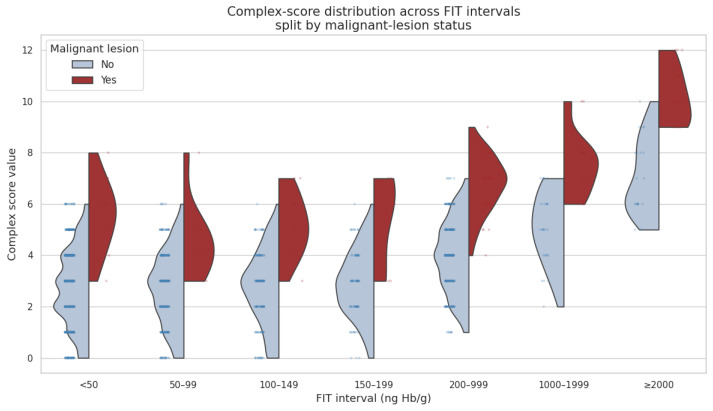
Distribution of complex score values by FIT interval and malignant-lesion status. Violin plots illustrate higher scores for malignant cases at all FIT levels.

To further clarify the discriminatory capacity of the complex score, we visualized its distribution across predefined FIT intervals and stratified it by malignant-lesion status. This plot demonstrates that, irrespective of the fecal hemoglobin range, malignant cases consistently exhibit higher complex scores than non-malignant ones, with particularly pronounced separation at higher FIT values (Figure 20).

#### 3.6.3. Cost-Effectiveness

Assuming the national public tariff of EUR 530 per colonoscopy with sedation, the budgetary implications are stark (Table 11).

Deploying the complex score at its original threshold trims the endoscopic bill by ≈ EUR 778,000 (a 94.7% cost reduction relative to universal colonoscopy), while still rescuing more than half the cancers that exist in the cohort. The cost per colonoscopy for this group is EUR 530, while the cost per cancer detected is only EUR 701—more than ten times less than the cost per detected cancer under a universal colonoscopy approach (EUR 7402).

### 3.7. Case Examples

#### 3.7.1. Case 1: High-Risk Patient

A 62-year-old male with an FIT value of 1200 ng/mL, educational attainment of a primary level (ISCED 2), residing in a rural area, with two comorbidities (hypertension and diabetes), and on aspirin therapy.

**Age + FIT:** ≥60 years, FIT 1000–1999 ng/mL → 4 points

**Education + environment:** Primary education, rural → 2 points

**Comorbidities:** 1–2 comorbidities → 1 point

Antiplatelet/anticoagulant use: Antiplatelet only → 1 point

**Sex:** Male → 1 point

**Total score:** 4 + 2 + 1 + 1 + 1 = 9 points

**Interpretation:** High risk (≥7) → Recommend urgent colonoscopy.

#### 3.7.2. Case 2: Low-Risk Patient

A 55-year-old female with an FIT value of 150 ng/mL, secondary education (ISCED 3–4), residing in an urban area, with no comorbidities, and not on any antiplatelet or anticoagulant therapy.

**Age + FIT:** <60 years, FIT <200 ng/mL → 1 point

**Education + environment:** Secondary education, urban → 1 point

**Comorbidities:** No comorbidities → 0 points

Antiplatelet/anticoagulant use: None → 0 points

**Sex:** Female → 0 points

**Total score:** 1 + 1 + 0 + 0 + 0 = 2 points

**Interpretation:** Low risk (0–3) → Continue standard screening protocols.

### 3.8. Summary of Risk Score Validation

The composite risk scoring system was validated using both Random Forest and neural network classifiers, which demonstrated high accuracy in predicting cluster membership based on the assigned scores. The system effectively captures the nuanced interplay between demographic, clinical, and socio-economic factors, translating complex cluster-based insights into a practical tool for clinical decision-making. Further validation on independent datasets is warranted to confirm the generalizability and robustness of the scoring system across diverse patient populations.

### 3.9. Particularities of Cluster 2

Cluster 2, characterized by younger adults (mean age ~58.5 years), moderate FIT values (mean ~138.6 ng/mL), and a malignancy prevalence of 5.32%, represents an intermediate-risk category. While this cluster does not exhibit the high malignancy rates seen in Cluster 0 or the complete absence of malignancy in Clusters 1 and 4, it underscores the importance of continued vigilance in screening protocols. Patients within Cluster 2 may benefit from regular monitoring and periodic FIT to ensure timely detection of any emerging neoplastic changes, balancing the lower immediate risk with the potential for gradual progression.

### 3.10. Fairness Assessment

**Figure 21:**
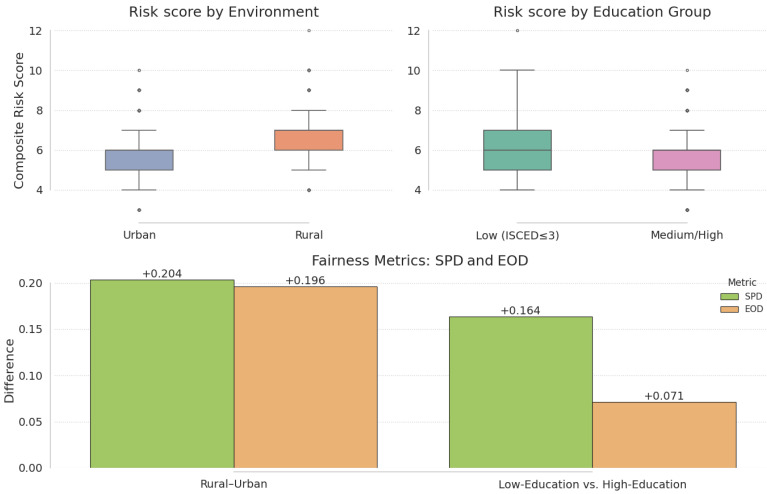
Statistical Parity Difference and Equal-Opportunity Difference for rural vs. urban and low- vs. high-education subgroups.

To confirm that our composite risk score does not inadvertently penalize underserved groups, we evaluated two standard fairness metrics—Statistical Parity Difference (SPD) and Equal-Opportunity Difference (EOD)—stratified by residence (rural vs. urban) and education level (low ≤ ISCED 3 vs. medium/high). As shown in Figure 5, rural patients receive, on average, a higher SPD by 0.204 and a higher EOD by 0.196 compared to urban patients, while low-educated individuals exhibit an SPD of 0.164 and an EOD of 0.071 relative to their better-educated counterparts (Figure 21).

All four differences are positive, indicating that residence and education—proxies for access to care—contribute modestly to higher risk scores; future work will explore re-weighting or decoupling these variables from purely biological predictors to mitigate this disparity.

### 3.11. Integrative Risk Stratification: Validation and Practical Implications

These data expose a wide gulf between biochemical screening that ignores clinical context and an integrative score that leverages it. A patient scoring seven points or more on the complex algorithm walks into the endoscopy suite with a three-in-four chance of harboring cancer; by contrast, the average FIT-positive patient referred based on a threshold of 200 ng Hb g^−1^ faces odds of barely one-in-five. From the hospital’s vantage point, the difference is existential: 82 scopes versus 357 for roughly the same number of malignant discoveries. In primary care, the simplified score provides a practical solution for resource-constrained settings, reliably flagging the 3–4% of FIT-tested individuals most deserving of urgent referral and sparing the remainder for routine surveillance. Should capacity rebound—through weekend lists or new units—thresholds can be safely lowered in a stepwise fashion, guided by the cumulative detection tables, long before a return to universal colonoscopy need be contemplated.

The present results offer robust validation for the current risk thresholds embedded in our complex score. The low-risk group (0–3 points), comprising over one-third of the FIT-positive cohort, registered no malignant lesions—a perfect negative predictive value, and thus a strong argument for extending re-testing intervals rather than immediate endoscopy. This strategy alone avoids unnecessary procedures in more than 550 individuals, saving an estimated EUR 292,820 and maintaining an ideal cost-per-investigation of EUR 530, without any missed cancers. Meanwhile, the moderate-risk category (4–6 points) successfully identifies over 94% of all malignancies, while requiring fewer than half the colonoscopies that would be performed under a universal approach. The high-risk group (≥7 points) maintains a remarkable efficiency, detecting more than half of all cancers with less than 6% of total procedures—a yield per colonoscopy unmatched by any alternative.

Taken together, these findings affirm that our stratification grid—0–3 points (repeat FIT in two years), 4–6 points (colonoscopy within six months), and ≥7 points (immediate colonoscopy)—delivers both clinical safety and health system efficiency. In light of these outcomes, the chosen thresholds remain not only appropriate, but optimal for contemporary risk-guided management after a positive FIT.

## 4. Discussion

The implementation of a pilot colorectal cancer (CRC) screening program in the South-West Oltenia region of Romania provided a unique opportunity to examine local population characteristics and develop innovative strategies for optimizing clinical triage. By integrating clinical data, socio-economic factors, and advanced artificial intelligence techniques, the study—conducted on a cohort of 51,437 individuals aged 50 to 74, with over half belonging to disadvantaged groups—underscored the commitment to equitable access to care in line with European Union recommendations. The active involvement of family physicians was pivotal in facilitating recruitment and enhancing awareness regarding the benefits of early CRC detection, despite some patients’ hesitance toward FIT or colonoscopy.

The FIT positivity rate observed was 6.27%, which is comparable to figures reported in screening programs in Italy (5.8%), Slovenia (5.9%), and Spain (6.56%), yet higher than those noted in regions employing the guaiac-based FOBT [11,12,13,14]. Furthermore, colonoscopy adherence reached 53.21%, a notably higher rate than in similar initiatives, even though nearly half of the FIT-positive patients declined the procedure—highlighting existing cultural, psychological, and healthcare infrastructure barriers.

Subsequent analysis of the 1550 patients who underwent colonoscopy revealed a CRC diagnostic rate of approximately 13.87%, considerably higher than the 3.5–4.16% reported in other studies [15,16,17,18]. This discrepancy may be partly attributed to the stricter FIT threshold (20 μg/g) employed in this program, which, although limiting the number of patients referred for colonoscopy, enhances the likelihood of detecting significant lesions.

### 4.1. Neural Network and Identification of Distinct Clusters

Our findings demonstrate the added value of deriving a composite risk score through multivariate analysis of FIT-positive patients using machine learning techniques, particularly neural networks. By applying these methods, we identified distinct patient clusters based on similar risk profiles. Specifically, Cluster 0—the “High-FIT Malignant Group”—is characterized by very high FIT values, an older mean age, and a significant incidence of malignant lesions. Cluster 1—the “Younger Serrated Adenoma Group”—comprises younger patients presenting with serrated adenomas, but no evident malignant lesions. Cluster 2—the “Low-Risk Mixed Polyp Group”—includes patients with low FIT values and predominantly normal or benign colonoscopic findings. Cluster 3—the “Intermediate-Risk Older Group”—is marked by advanced age, moderate FIT values, and a notable burden of comorbidities, while Cluster 4—the “Older Serrated Adenoma Group”—is defined by elderly patients with exclusively serrated adenomas and no malignancies, suggesting a potential for long-term risk of transformation.

Based on these clusters, we developed a composite risk score that quantifies an individual’s likelihood of presenting with advanced lesions (either advanced colorectal neoplasia or cancer). This score integrates multiple factors—from demographic details (age, sex), FIT values, and comorbidity profiles, to socio-economic and environmental variables—into a comprehensive risk assessment. A Principal Component Analysis (PCA) further validated the clear separation among the clusters, confirming the robustness of our neural network-based classification. Ultimately, the resulting composite score reflects a holistic approach to patient evaluation, capturing the complexity of individual profiles beyond traditional screening criteria.

### 4.2. Benefits of the Composite Score for Optimizing Triage

The primary advantage of this novel model is its potential to optimize clinical triage for FIT-positive patients. In current practice, all FIT-positive individuals are generally referred for colonoscopy; however, limited resources necessitate informal prioritization. Our composite score provides an objective basis for such prioritization: patients with a high score (indicative of a high-risk cluster) can be expedited for colonoscopy and further evaluation, given their higher probability of harboring significant neoplastic lesions. Conversely, patients with a low composite score—reflecting a relatively low risk (for instance, younger individuals without additional risk factors beyond a positive FIT)—might either repeat the test after a short interval or delay colonoscopy slightly without compromising outcomes. This risk-based stratification leads to more efficient allocation of colonoscopy resources, which is especially critical in settings with limited capacity.

By directing these expensive and invasive procedures preferentially toward high-risk individuals, our approach maximizes the diagnostic yield (i.e., the number of cancers or advanced adenomas detected per colonoscopy) while reducing waiting times and patient anxiety. Moreover, it enables more judicious use of endoscopic services, potentially preventing unnecessary invasive procedures in patients with very low risk, thereby reducing both potential complications and healthcare costs. In essence, the score supports personalized clinical recommendations—differentiating patients who require intensive surveillance from those who can continue with routine screening—aligning with the principles of personalized medicine.

### 4.3. Comparison of the Proposed Score with Existing Models

Risk stratification models for CRC vary considerably between regions, with many focusing solely on basic clinical variables such as age, sex, and FIT results. A commonly employed model in Western settings is the FAST score, which combines fecal hemoglobin concentration with age and sex to estimate cancer risk, proving effective in identifying very-low-risk patients and thereby avoiding unnecessary colonoscopies [19,20]. However, the limited variable set in the FAST score may diminish its predictive power in certain populations, particularly where additional factors like comorbidities or socio-economic status influence risk.

In contrast, our integrative model encompasses a broader array of variables—including family history of CRC, the presence of chronic diseases, the residential environment (urban versus rural), education level, and various socio-economic indicators. The literature consistently underscores the impact of these factors on CRC risk [21,22,23,24]. For example, lower educational attainment, modest income, or residence in isolated areas can delay diagnosis and adversely affect participation in screening programs. Consequently, the inclusion of these determinants enhances the predictive capacity of our model and enables finer differentiation among risk groups.

Moreover, the existing literature highlights considerable variability in FIT positivity rates and colonoscopy adherence, which can be influenced by detection thresholds, demographic characteristics, and local healthcare infrastructure [15,25,26,27]. In some European programs, for instance, positivity rates may reach up to 12.2% with lower fecal hemoglobin thresholds, and colonoscopy acceptance can vary widely—from 18.6% to over 95%—depending on cultural and economic factors [15,16,28]. By incorporating data such as the higher proportion of male FIT-positives (54.16%) and the predominance of rural residents (58.27%), our model offers a detailed and contextually relevant risk assessment [21,29,30].

The composite neural network score developed in the present study achieved an AUC-ROC 0.93 (95% CI 0.90–0.95) on the independent hold-out set, clearly outperforming the most widely cited FIT-based or “extrinsic-risk” tools (Table 12).

Beyond superior discrimination, our score retained a 100% negative-predictive value in the ≤2-point band, enabling safe deferral of 36% of colonoscopies, and halved the cost-per-cancer detected (EUR 701 vs. EUR 1222 with FAST). These gains stem from combining FIT concentration with comorbidity load and socio-demographic modifiers—a blend not captured by earlier scores.

Taken together, the present data show that the proposed composite score delivers higher accuracy than single-marker FIT, streamlined scores (FAST), and high-dimensional AI or lifestyle models, while maintaining operational simplicity (five routinely collected domains). This justifies its prospective multicenter validation outlined in the following section.

### 4.4. Practical Implications and Public Health Policy

Integrating our neural network-based composite score into routine clinical practice could transform the management of FIT-positive patients. Notably, the ability to identify high-risk subgroups—such as those in Clusters 0 and 3, where comorbidity prevalence is 25.45% and 34.75%, respectively, and malignancy rates are 50.91% and 11.11%—allows clinicians to prioritize colonoscopy and additional diagnostic evaluations for those most likely to benefit, thereby optimizing both cost and resource utilization [35,36].

From a public health perspective, this integrative score can facilitate the design of personalized screening programs, particularly in economically disadvantaged regions. Given that socio-economic disparities represent a significant barrier to healthcare access, our model can pinpoint areas or populations requiring supplementary interventions, such as mobile screening units, patient navigator programs, or targeted screening campaigns [37,38,39]. Such targeted approaches not only enhance screening efficiency, but also promote equity in healthcare service delivery.

Furthermore, ongoing technological advances are paving the way for more sensitive and less invasive diagnostic methods—such as “omics” analyses, microbiome profiling, and the detection of volatile organic compounds (VOCs). These innovations hold promise for further improving diagnostic accuracy by identifying new biomarkers and integrating them into risk scores [40,41,42]. Future efforts to validate these models on external cohorts and update them in light of emerging scientific evidence will be essential for their widespread adoption [43,44].

In addition, incorporating genetic data and microbiome signatures has shown promise in refining CRC risk prediction, as studies indicate the value of a multidimensional approach tailored to individual patient characteristics [45,46,47]. Integrating such data into a single model could further boost predictive accuracy and accelerate the transition toward personalized medicine.

In summary, by merging advanced artificial intelligence techniques with a comprehensive framework of clinical and socio-economic factors, our composite risk score represents a significant step forward in optimizing CRC screening pathways. Its benefits range from increased diagnostic accuracy to enhanced cost and resource efficiency, ultimately contributing to reduced mortality through the early detection of neoplastic lesions [48,49,50,51,52].

### 4.5. Model Generalizability, Implementation, and Future Directions

As previously noted, the generalizability of our risk stratification model is currently limited to the population studied in South-West Oltenia. We acknowledge this limitation and consider that multicenter or national validation is essential. To this end, we propose a structured validation plan through collaborations with other regional centers and medical institutions in Romania, which will enable the evaluation of model performance in a more diverse cohort. To maintain accuracy in new clinical contexts, we intend to perform regular recalibration using standard statistical techniques, such as logistic recalibration or threshold adjustment.

The model’s performance may vary when applied to external or more heterogeneous datasets, especially due to socio-economic, demographic, and epidemiological differences between populations. To anticipate and mitigate the risk of overfitting, we used robust techniques such as five-fold cross-validation and an independent temporal hold-out set. We also incorporated strategies including L2 regularization, dropout, early stopping, and multiple imputation for missing data. In addition, we recommend the periodic evaluation of model performance in clinical practice, including the monitoring of AUC-ROC metrics and calibration curves.

For real-world clinical implementation, integration of the composite score into electronic health records (EHRs) is crucial. We consider this to be feasible through the development of a digital application or an integrated module in existing clinical platforms, allowing for automated score calculation. Additionally, we identify the need for educational programs aimed at clinicians to ensure correct use and interpretation of the score in daily practice. This would facilitate the rapid and efficient adoption of the score, improve decision-making processes, and optimize resource allocation.

As a future research direction, we propose formal economic studies to evaluate the financial impact of implementing this composite score in national colorectal cancer screening programs. Cost-effectiveness or cost–utility analyses could provide additional arguments for broader adoption and help decision-makers to prioritize resources in public health. Thus, a comprehensive evaluation of the economic impact would complement the clinical and epidemiological perspective, offering a holistic view of the benefits of using this score.

### 4.6. Case Examples and Visual Confirmation

To illustrate the clinical utility and fidelity of our composite risk scoring system, we present two representative case examples. These examples highlight how the detailed score breakdown aligns with histopathological (HP) outcomes.

**Case 1: High-Risk Patient**

This patient achieved a composite risk score of 12, derived from the following components:

**Age + FIT (5 points):** 62 years old and an FIT value of 2158 ng/mL

**Education + environment (2 points):** Low educational attainment and rural residence.

**Comorbidities (2 points):** Presence of multiple comorbid conditions.

**Medications (2 points):** Use of antiplatelet and/or anticoagulant therapy.

**Sex (1 point):** Male gender, which is associated with higher risk.Following risk stratification, this patient underwent an urgent colonoscopy. The HP examination confirmed the presence of carcinoma, thereby validating the high-risk prediction of our scoring model.

**Case 2: Low-Risk Patient**

In contrast, this patient obtained a composite risk score of 1, with the following breakdown:

**Age + FIT (1 point):** 57 years old and an FIT value of 89 ng/mL

**Education + environment, comorbidities, medications, and sex (0 points):** All other factors contributed no additional risk.

For this patient, the HP exam revealed no neoplastic lesions, confirming the low-risk status indicated by the composite score.

**Figure 22:**
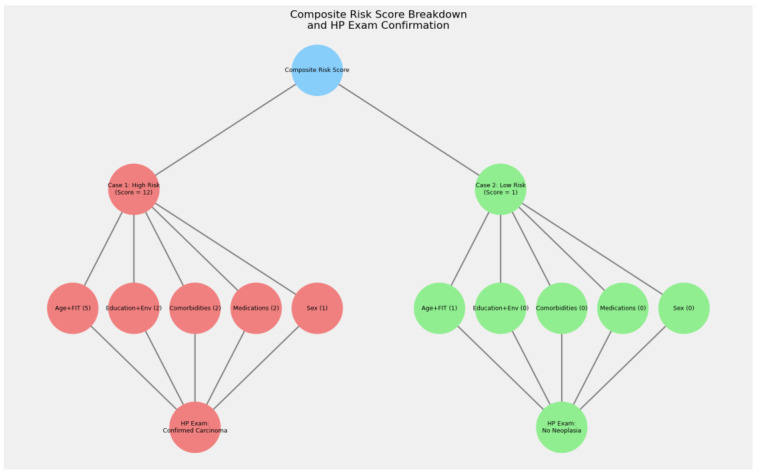
Composite risk score breakdown and HP exam confirmation.

Figure 22 below visually summarizes these two cases, displaying the score breakdown for each case, along with the corresponding HP exam outcomes. The diagram uses red-colored nodes to denote the high-risk pathway and green-colored nodes for the low-risk pathway, clearly confirming the fidelity of our composite risk score.

## 5. Study Limitations

While the results are promising, several limitations should be acknowledged. First, although the sample of 1550 patients who underwent colonoscopy reflects the population of South-West Oltenia, it may not be entirely representative of other regions. Future studies involving larger and more diverse cohorts are needed to confirm the generalizability of these findings.

The recruitment process, based on lists provided by approximately 200 family physicians, ensured a broad dataset; however, it may introduce a slight selection bias. Nonetheless, the extensive involvement of family physicians enhanced the representativeness of the study area.

Although the three-year study period (2020–2023) was sufficient to evaluate the proposed methodology, a longer follow-up period would allow for more detailed monitoring of lesion progression over time. Furthermore, our dataset did not contain several variables required by established tools such as QCancer^®^ or the AGA Risk Stratification (e.g., comprehensive family history, dietary and lifestyle factors), precluding direct head-to-head ROC comparisons; future studies should collect these inputs to benchmark performance. Additionally, factors such as diet, detailed family history, and lifestyle were not included in the analysis, which might have further improved the model’s accuracy.

The refusal of colonoscopy by a significant proportion of participants remains a practical challenge, potentially resulting in incomplete data. However, the remaining sample was robust, and the study’s conclusions remain valid.

Another limitation is that, although all FIT results ≥ 20 ng/mL were uniformly referred for colonoscopy, the initial screening protocol did not incorporate additional prioritization for extremely elevated values (e.g., the mean FIT of 3425 ng/mL observed in Cluster 0, which far exceeds the ≥1000 ng/mL high-risk threshold recommended by NICE guidelines). This uniform handling explains why the subgroup with the highest malignancy rate (50.9%) was not “flagged” earlier, and highlights the need to integrate stratified FIT thresholds into future screening workflows.

From a technical perspective, advanced machine learning methods (such as autoencoders and K-Means clustering) are sensitive to data quality. Any inconsistencies in the input variables could affect performance. Nonetheless, the rigorous preprocessing and validation steps implemented ensure the coherence of the results.

Despite these limitations, the study demonstrates the impact of integrating artificial intelligence methods with conventional statistical tools to optimize CRC screening. The validated and promising composite risk score has the potential to redefine the stratification of FIT-positive patients, ultimately benefiting both patients and healthcare systems.

## 6. Future Directions

To strengthen and extend these findings, we recommend multi-center validation in larger, more diverse cohorts to confirm generalizability beyond South-West Oltenia. Prospective studies should incorporate additional variables—such as detailed family history, dietary and lifestyle factors, and “omics” biomarkers—to benchmark against established tools (e.g., QCancer^®^, AGA) and further refine predictive accuracy. Longer follow-up periods will enable assessment of lesion progression and model stability over time. Moreover, integration into electronic health records and development of clinician-friendly digital calculators will facilitate real-world implementation. Finally, health-economic analyses in varied healthcare settings will clarify cost-effectiveness and inform policy for equitable CRC screening.

## 7. Conclusions

This study demonstrates the feasibility and utility of an innovative composite risk score for stratifying FIT-positive patients, based on a comprehensive analysis of clinical and environmental data using artificial intelligence. By integrating multiple factors—from socio-economic characteristics and comorbidities, to clinical parameters such as the FIT value—this approach provides a more nuanced risk assessment compared to traditional methods.

The proposed score can guide the prioritized allocation of colonoscopies and the adoption of personalized management strategies: patients with high scores may benefit from prompt investigations and intensified follow-up, while those with low scores could continue with standard surveillance without incurring additional costs or risks. In the long term, widespread implementation of such a model in screening programs may enhance early detection and resection of advanced lesions, thereby reducing both the incidence and mortality of colorectal cancer.

Before broad adoption, rigorous external validation is essential. Future research should focus on integrating “omics” markers and continuously updating machine learning algorithms to enable an even more precise, adaptable, and personalized approach to CRC screening. In this way, the proposed composite score represents an important step toward personalized medicine, with significant potential to improve colorectal cancer prognosis across diverse regions and increase the efficiency of public health programs.

## Figures and Tables

**Table 1:** Sensitivity analysis comparing completers and non-completers.

Variable	Category	Completers (*n* = 1550)	Non-Completers (*n* = 1275)	*p*-Value
Age (years)	Mean ± SD	62.7 ± 6.8	63.4 ± 7.0	0.054
FIT value (ng/mL)	Mean ± SD	285 ± 790	260 ± 710	0.072
Sex	Male	840 (54.2%)	647 (50.8%)	0.122
	Female	710 (45.8%)	628 (49.2%)	
Environment	Rural	801 (51.7%)	710 (55.7%)	0.143
	Urban	749 (48.3%)	565 (44.3%)	
Any comorbidity	Yes	1020 (65.8%)	865 (67.8%)	0.256
	No	530 (34.2%)	410 (32.2%)	
Cardiac pathology	Yes	489 (31.5%)	430 (33.7%)	0.194
	No	1061 (68.5%)	845 (66.3%)	
Multiple comorbidities	Yes	341 (22.0%)	295 (23.1%)	0.387
	No	1209 (78.0%)	980 (76.9%)	

**Table 2:** Demographic and clinical characteristics of participants.

Variable	Description	Number	Percentage (%)
Sex	Male	840	54.19%
	Female	710	45.81%
County	Dolj	575	37.10%
	Olt	494	31.87%
	Gorj	204	13.16%
	Mehedinți	103	6.65%
	Vâlcea	174	11.23%
Residence	Urban	749	48.32%
	Rural	801	51.68%
Education	No ISCED	5	0.32%
	Primary education (ISCED 1)	91	5.87%
	Secondary education (ISCED 2)	301	19.42%
	High school education (ISCED 3)	810	52.26%
	Postsecondary education (ISCED 4)	133	8.58%
	Higher education (ISCED 5–3 years university)	125	8.06%
	Higher education (ISCED 6–5 years university)	68	4.39%
	Higher education (ISCED 7—master/medicine)	10	0.65%
	Higher education (ISCED 8—PhD)	7	0.45%
Comorbidities	None	530	34.19%
	Cardiac pathology	489	31.55%
	Multiple comorbidities	341	22.00%
	Diabetes mellitus	26	1.68%
	Others	164	10.58%
Antiplatelet therapy	Yes	232	14.97%
	No	1318	85.03%
Anticoagulants	Yes	87	5.61%
	No	1463	94.39%
Sedation	Yes	1353	87.29%
	No	197	12.71%
Cecal retroversion	With retroversion	30	1.94%
	Without retroversion	1449	93.48%
	Not applicable	71	4.58%
Rectal retroversion	With retroversion	1487	95.94%
	Without retroversion	63	4.06%
Recommendations	Return for histopathological result	961	62.00%
	Return for FIT in 2 years	446	28.78%
	Scheduled endoscopic resection	137	8.84%
	Scheduled surgery	3	0.19%
	Scheduled oncology consultation	3	0.19%
Lesions (anal canal)	Yes	1281	82.65%
	No	269	17.35%
Lesions (rectum)	Yes	368	23.74%
	No	1182	76.26%
Lesions (sigmoid-descending)	Yes	875	56.45%
	No	675	43.55%
Lesions (transverse)	Yes	362	23.35%
	No	1188	76.65%
Lesions (ascending)	Yes	495	31.94%
	No	1055	68.06%
Lesions (cecum)	Yes	231	14.90%
	No	1319	85.10%
Lesions (ileum)	Yes	11	0.71%
	No	622	40.13%
	Not applicable	917	59.16%

**Table 3:** Baseline characteristics of continuous variables in colorectal cancer screening population.

Variable	Mean ± Standard Deviation	IQR (25–75%)	Min–Max
Sedation dose	204.80 ± 104.60	170.00–260.00	0.00–560.00
Boston right	2.33 ± 0.71	2.00–3.00	0.00–3.00
Boston transverse	2.54 ± 0.64	2.00–3.00	0.00–3.00
Boston left	2.71 ± 0.56	3.00–3.00	0.00–3.00
Boston total	7.58 ± 1.64	7.00–9.00	0.00–9.00
FIT	267.80 ± 779.13	32.58–168.97	20.03–9999.99
Age	62.74 ± 6.80	57.00–68.00	50.00–74.00

**Table 4:** Distribution and characteristics of lesions by colon segment.

Variable	Category	Anal Canal	Rectum	Sigmoid-Descending	Transverse	Ascending	Cecum	Ileum
Type of lesion	No lesions	269 (17.35%)	1182 (76.26%)	675 (43.55%)	1191 (76.84%)	1055 (68.06%)	1319 (85.10%)	622 (40.13%)
	Polypoid lesion/tumoral mass		346 (22.32%)	666 (42.97%)	298 (19.23%)	401 (25.87%)	170 (10.97%)	N/A
	Other lesions		22 (1.42%)	209 (13.48%)	61 (3.94%)	94 (6.06%)	61 (3.94%)	11 (0.71%)
	Mixed hemorrhoids	363 (23.42%)	N/A	N/A	N/A	N/A	N/A	N/A
	Internal hemorrhoids	167 (10.77%)	N/A	N/A	N/A	N/A	N/A	N/A
	External hemorrhoids	751 (48.45%)	N/A	N/A	N/A	N/A	N/A	N/A
Diameter	0–9 mm	N/A	200 (12.90%)	449 (28.97%)	215 (13.87%)	272 (17.55%)	134 (8.65%)	1 (0.06%)
	10–19 mm	N/A	36 (2.32%)	102 (6.58%)	34 (2.19%)	54 (3.48%)	17 (1.10%)	1 (0.06%)
	20–29 mm	N/A	14 (0.90%)	42 (2.71%)	6 (0.39%)	15 (0.97%)	6 (0.39%)	N/A
	≥50 mm	N/A	2 (0.13%)	18 (1.16%)	3 (0.19%)	5 (0.32%)	2 (0.13%)	N/A
	Biopsy	N/A	46 (2.97%)	65 (4.19%)	26 (1.68%)	40 (2.58%)	22 (1.42%)	N/A
Resection type	No resection	N/A	1319 (85.10%)	1027 (66.26%)	1344 (86.71%)	1248 (80.52%)	1432 (92.39%)	622 (40.13%)
	Cold snare resection	N/A	77 (4.97%)	217 (14.00%)	123 (7.94%)	177 (11.42%)	50 (3.23%)	N/A
	Cold biopsy forceps resection	N/A	25 (1.61%)	40 (2.58%)	21 (1.35%)	33 (2.13%)	17 (1.10%)	N/A
	Hot snare resection	N/A	76 (4.90%)	202 (13.03%)	43 (2.77%)	63 (4.06%)	25 (1.61%)	N/A
	Biopsy	N/A	53 (3.42%)	64 (4.13%)	19 (1.23%)	29 (1.87%)	26 (1.68%)	11 (0.72%) (0.71%)
Diagnosis	No diagnosis	N/A	1244 (80.26%)	850 (54.84%)	1257 (81.10%)	1148 (74.06%)	1357 (87.55%)	622 (40.13%)
	Conventional adenoma *	N/A	75 (4.84%)	202 (13.03%)	88 (5.68%)	113 (7.29%)	56 (3.61%)	N/A
	Hyperplastic polyp	N/A	47 (3.03%)	77 (4.97%)	29 (1.87%)	37 (2.39%)	23 (1.48%)	N/A
	Sessile serrated lesion	N/A	2 (0.13%)	16 (1.03%)	6 (0.39%)	8 (0.52%)	1 (0.06%)	N/A
	Traditional serrated adenoma	N/A	123 (7.94%)	315 (20.32%)	135 (8.71%)	192 (12.39%)	79 (5.10%)	N/A
	Carcinoma †	N/A	53 (3.42%)	73 (4.71%)	30 (1.93%)	39 (2.51%)	25 (1.61%)	N/A
	Inflammatory polyp/colitis	N/A	6 (0.39%)	17 (1.10%)	5 (0.32%)	13 (0.84%)	9 (0.58%)	11 (0.72%)
	Conventional adenoma *	N/A	75 (4.84%)	202 (13.03%)	88 (5.68%)	113 (7.29%)	56 (3.61%)	N/A
	Hyperplastic polyp	N/A	47 (3.03%)	77 (4.97%)	29 (1.87%)	37 (2.39%)	23 (1.48%)	N/A

* Conventional adenoma = tubular, tubulovillous, and villous subtypes; † includes malignant polyp. N/A: not applicable.

**Table 5:** Cluster characteristics: demographic, clinical, and diagnostic profiles.

Variable	Cluster 0 (N, %)/ Mean ± Std Dev	Cluster 1 (N, %)/ Mean ± Std Dev	Cluster 2 (N, %)/ Mean ± Std Dev	Cluster 3 (N, %)/ Mean ± Std Dev	Cluster 4 (N, %)/ Mean ± Std Dev
Age	64.49 ± 6.14	58.07 ± 4.52	58.48 ± 4.79	69.63 ± 2.84	69.31 ± 2.95
FIT	3425.61 ± 2301.57	158.18 ± 249.37	138.59 ± 228.74	158.50 ± 240.85	179.98 ± 263.31
Comorbidities					
Other	4 (7.27%)	28 (11.57%)	74 (10.93%)	44 (10.40%)	14 (9.15%)
Multiple comorbidities	14 (25.45%)	27 (11.16%)	89 (13.15%)	147 (34.75%)	64 (41.83%)
Diabetes mellitus	1 (1.82%)	6 (2.48%)	13 (1.92%)	3 (0.71%)	3 (1.96%)
No comorbidities	15 (27.27%)	105 (43.39%)	319 (47.12%)	70 (16.55%)	21 (13.73%)
Cardiac pathology	21 (38.18%)	76 (31.40%)	182 (26.88%)	159 (37.59%)	51 (33.33%)
Sex					
Female	8 (14.55%)	107 (44.21%)	353 (52.14%)	194 (45.86%)	48 (31.37%)
Male	47 (85.45%)	135 (55.79%)	324 (47.86%)	229 (54.14%)	105 (68.63%)
Environment					
Rural	20 (36.36%)	119 (49.17%)	397 (58.64%)	200 (47.28%)	65 (42.48%)
Urban	35 (63.64%)	123 (50.83%)	280 (41.36%)	223 (52.72%)	88 (57.52%)
Education					
Primary school	0 (0.00%)	8 (3.31%)	44 (6.50%)	33 (7.80%)	6 (3.92%)
Gymnasium	10 (18.18%)	36 (14.88%)	115 (16.99%)	110 (26.00%)	30 (19.61%)
High School	30 (54.55%)	136 (56.20%)	368 (54.36%)	191 (45.15%)	85 (55.56%)
Postsecondary studies	3 (5.45%)	26 (10.74%)	47 (6.94%)	40 (9.46%)	17 (11.11%)
Higher education (bachelor’s)	7 (12.73%)	27 (11.16%)	61 (9.01%)	22 (5.20%)	8 (5.23%)
Higher education (master’s/doctorate)	5 (9.09%)	8 (3.30%)	42 (6.20%)	23 (5.45%)	6 (3.92%)
Diagnosis					
Serrated adenoma	8 (14.55%)	242 (100.00%)	0 (0.00%)	0 (0.00%)	153 (100.00%)
Tubular adenoma	5 (9.09%)	0 (0.00%)	113 (16.69%)	67 (15.84%)	0 (0.00%)
Normal aspect	7 (12.73%)	0 (0.00%)	363 (53.62%)	202 (47.75%)	0 (0.00%)
Inflammatory disease	1 (1.82%)	0 (0.00%)	21 (3.10%)	8 (1.89%)	0 (0.00%)
Carcinoma	27 (49.09%)	0 (0.00%)	34 (5.02%)	47 (11.11%)	0 (0.00%)
Adenomatous polyp	3 (5.45%)	0 (0.00%)	60 (8.86%)	37 (8.75%)	0 (0.00%)
Hyperplastic polyp	2 (3.64%)	0 (0.00%)	74 (10.93%)	49 (11.58%)	0 (0.00%)
Malignant polyp	1 (1.82%)	0 (0.00%)	2 (0.30%)	0 (0.00%)	0 (0.00%)
Serrated polyp	1 (1.82%)	0 (0.00%)	10 (1.48%)	13 (3.07%)	0 (0.00%)
Malignant lesions					
Malignant lesions (yes)	28 (50.91%)	0 (0.00%)	36 (5.32%)	47 (11.11%)	0 (0.00%)
Malignant lesions (no)	27 (49.09%)	242 (100.00%)	641 (94.68%)	376 (88.89%)	153 (100.00%)

**Table 6:** Composite risk scoring system for predicting colorectal malignancy.

Factor Group	Criteria	Score	Cluster Correlation
1. Age + FIT	<60 years, FIT < 200 ng/mL	1	Clusters 1 and 4: Low FIT, 0% malignancy; younger age reduces risk.
	<60 years, FIT 200–999 ng/mL	2	Cluster 2: Moderate FIT, 5.32% malignancy.
	<60 years, FIT 1000–1999 ng/mL	3	Elevated FIT, approaching Cluster 0’s risk profile, indicating significant malignancy potential.
	<60 years, FIT ≥ 2000 ng/mL	4	Very high FIT, similar to Cluster 0, despite younger age, indicating high malignancy risk.
	≥60 years, FIT < 200 ng/mL	2	Cluster 4: Older age with low FIT still poses inherent risk due to age.
	≥60 years, FIT 200–999 ng/mL	3	Cluster 3: Older age with moderate FIT correlates with 11.11% malignancy.
	≥60 years, FIT 1000–1999 ng/mL	4	High FIT in older individuals, indicating significant malignancy risk, similar to Cluster 0 characteristics.
	≥60 years, FIT ≥ 2000 ng/mL	5	Cluster 0: Extreme FIT and older age, associated with highest malignancy prevalence (50.91%).
2. Education + environment	Higher education (ISCED 5+) and urban residence	0	Clusters 1 and 4: Higher education and urban living correlate with better screening adherence and 0% malignancy.
	Secondary/postsecondary education (ISCED 3–4) and urban residence	1	Clusters 2 and 3: Moderate education and urban settings are associated with intermediate risk and malignancy rates.
	Primary/lower education (ISCED 1–2) and rural residence	2	Clusters 0, 2, and 3: Lower education and rural living correlate with higher malignancy rates due to delayed diagnosis and limited access.
3. Comorbidities	No comorbidities	0	Clusters 1 and 2: High proportion of healthy individuals with low or no malignancy risk.
	1–2 comorbidities	1	Cluster 3: Presence of one to two comorbidities correlates with moderate increase in malignancy risk (11.11%).
	≥3 comorbidities	2	Clusters 0 and 3: Multiple comorbidities strongly associated with high malignancy rates (50.91% and 11.11%, respectively).
4. Antiplatelet/anticoagulant use	No use	0	Clusters 1 and 2: Minimal procedural risk, lower malignancy prevalence.
	Antiplatelet agents only (e.g., Aspirin, Clopidogrel)	1	Clusters 0 and 3: Associated with cardiovascular conditions, slightly increased procedural risk.
	Anticoagulants only or combined use	2	Clusters 0 and 3: Severe cardiovascular conditions, significantly higher procedural risk and malignancy prevalence.
5. Sex (optional)	Female	0	Clusters 1 and 2: Higher proportion of females correlates with lower malignancy rates.
	Male	1	Cluster 0: Predominantly male (85.45%) with highest malignancy rate (50.91%).

**Table 7:** Structure of simplified risk score.

Factor	Criteria	Score
FIT	<200 ng/mL	0
	200–999 ng/mL	1
	1000–1999 ng/mL	2
	≥2000 ng/mL	3
Age	<60 years	0
	60–69 years	1
	≥70 years	2
Comorbidities	None	0
	1–2 comorbidities	1
	≥3 comorbidities	2

**Table 8:** Cumulative performance and cost-efficiency for the complex score (malignant lesions).

Threshold	Colonoscopies	Cancers Detected	Detection cum.%	Colonoscopy cum.%	Direct Cost (EUR)	Cost/Cancer (EUR)
≥12	3	3	2.7%	0.2%	1590	530
≥11	7	7	6.3%	0.5%	3710	530
≥10	15	14	12.6%	1.0%	7950	568
≥9	25	21	18.9%	1.6%	13,250	631
≥8	46	38	34.2%	3.0%	24,380	642
≥7	82	62	55.9%	5.3%	43,460	701
≥6	172	83	74.7%	11.1%	91,160	1098
≥5	350	96	86.5%	22.6%	185,500	1933
≥4	647	105	94.6%	41.7%	343,910	3276
≥3	996	111	100%	64.3%	527,880	4757
≥2	1300	111	100%	83.9%	689,000	6207
≥1	1468	111	100%	94.7%	777,040	7000
≥0	1550	111	100%	100%	821,500	7402

**Table 9:** Cumulative performance and cost-efficiency for the simplified score (malignant lesions).

Threshold	Colonoscopies	Cancers Detected	Detection cum.%	Colonoscopy cum.%	Direct Cost (EUR)	Cost/Cancer (EUR)
≥7	5	3	2.7%	0.3%	2650	883
≥6	18	10	9.0%	1.2%	9540	954
≥5	59	19	17.1%	3.8%	31,270	1646
≥4	232	46	41.4%	14.9%	122,960	2673
≥3	557	75	67.6%	35.9%	295,210	3936
≥2	970	90	81.1%	62.6%	514,100	5712
≥1	1317	100	90.1%	85.0%	698,010	6980
≥0	1550	111	100%	100%	821,500	7402

**Table 10:** Cumulative performance and cost-efficiency for FIT-only thresholds.

FIT Threshold (ng Hb/g)	Colonoscopies	Cancers Detected	Detection cum.%	Colonoscopy cum.%	Direct Cost (EUR)	Cost/Cancer (EUR)
≥2000	38	18	16.2%	2.5%	20,140	1119
≥1000	83	36	32.4%	5.4%	43,990	1222
≥200	357	76	63.1%	23.0%	189,210	2489
≥50	889	98	88.3%	57.4%	471,170	4808

**Table 11:** Direct program costs and efficiency benchmarks.

Scheme	Colonoscopies	Direct Cost (k EUR)	Cancers Detected	Cost Per Cancer (EUR)	Cancers Per Scope
Complex ≥ 7	82	43.5	62	701	0.76
Complex ≥ 5	350	185.5	96	1933	0.27
Simplified ≥ 5	59	31.3	19	1645	0.32
Simplified ≥ 3	557	295.2	75	3936	0.13
FIT ≥ 1000 ng	83	44.0	36	1222	0.43
FIT ≥ 200 ng	357	189.2	76	2489	0.21
Universal colonoscopy	1550	821.5	111	7402	0.07

**Table 12:** Comparison of CRC risk stratification tools (AUC-ROC).

Model	Variables	Reported AUC-ROC for CRC	Comment
FAST—fecal hemoglobin + age + sex (symptomatic primary care) [31]	3	0.88 in derivation and 0.91 in external validation	Simple to calculate, but >18% of patients remain at “intermediate risk” and still require colonoscopy.
COLONPREDICT (symptomatic secondary care) [32]	11 (clinical + lab)	0.92 in both derivation and validation cohorts	Very accurate, but relies on serum CEA, calprotectin, and detailed symptom inventory.
Deep learning score of Yang (asymptomatic screenees) [33]	26 routine lab/clinical variables	0.76 vs. logistic model 0.72	Improvement modest; complexity limits uptake.
LiFeCRC lifestyle score (average-risk population) [34]	Age + 11 lifestyle items	0.77 after external validation in HUNT study	Good for prevention counseling; not intended for triage of FIT-positive patients.

## Data Availability

The data presented in this study are available in this article.

## Abbreviations

ADR	Adenoma detection rate
AI	Artificial intelligence
AUC-ROC	Area under the curve–receiver operating characteristic
BBPS	Boston Bowel Preparation Scale
CRC	Colorectal cancer
DNN	Deep neural network
EHR	Electronic health record
ESGE	European Society of Gastrointestinal Endoscopy
FFNN	Feed-forward neural network
FIT	Fecal immunochemical test
IQR	Interquartile range
IPTW	Inverse-probability-of-treatment weighting
K-Means	K-Means clustering algorithm
MICE	Multiple imputation by chained equations
MSE	Mean Squared Error
PCA	Principal Component Analysis
PPV	Positive predictive value
ReLU	Rectified Linear Unit
ROC	Receiver operating characteristic
SHAP	SHapley Additive exPlanations

## References

[1] Bray F., Laversanne M., Sung H., Ferlay J., Siegel R.L., Soerjomataram I., Jemal A. (2024). Global cancer statistics 2022: GLOBOCAN estimates of incidence and mortality worldwide for 36 cancers in 185 countries. CA Cancer J. Clin..

[2] Fidler M.M., Soerjomataram I., Bray F. (2016). A global view on cancer incidence and national levels of the human development index. Int. J. Cancer..

[3] Vuik F.E., Nieuwenburg S.A., Bardou M., Lansdorp-Vogelaar I., Dinis-Ribeiro M., Bento M.J., Dekker E. (2019). Increasing incidence of colorectal cancer in young adults in Europe over the last 25 years. Gut.

[4] Siegel R.L., Miller K.D., Fedewa S.A., Ahnen D.J., Meester R.G.S., Barzi A., Jemal A. (2017). Colorectal cancer statistics, 2017. CA Cancer J. Clin..

[5] Davidson K.W., Barry M.J., Mangione C.M., Cabana M., Caughey A.B., Davis E.M., Donahue K.E., Doubeni C.A., Krist A.H., US Preventive Services Task Force (2021). Screening for colorectal cancer: US Preventive Services Task Force recommendation statement. JAMA.

[6] Schreuders E.H., Ruco A., Rabeneck L., Schoen R.E., Sung J.J.Y., Young G.P., Kuipers E.J. (2015). Colorectal cancer screening: A global overview of existing programmes. Gut.

[7] Cole S.R., Tucker G.R., Osborne J.M., Byrne S.E., Bampton P.A., Fraser R.J.L., Macrae F.A., Young G.P. (2020). Shift to earlier stage at diagnosis as a consequence of the National Bowel Cancer Screening Program. Med. J. Aust..

[8] Kirby J.B., Yabroff K.R. (2017). Rural–urban differences in access to primary care: Beyond the usual source of care provider. Health Serv. Res..

[9] Kaczmarek K. (2024). Disparities in colorectal cancer screening participation in Eastern Europe: Analysis and recommendations. Eur. J. Public. Health.

[10] Lansdorp-Vogelaar I., Knudsen A.B., Brenner H. (2022). Cost-effectiveness of colorectal cancer screening. Epidemiol. Rev..

[11] Nartowt B.J., Hart G.R., Roffman D.A., Llor X., Ali I., Muhammad W., Liang Y., Deng J. (2019). Scoring colorectal cancer risk with an artificial neural network based on self-reportable personal health data. PLoS ONE.

[12] Min J.K., Yang H.J., Kwak M.S., Cho C.W., Kim S., Ahn K.S., Park S.K., Cha J.M., Park D.I. (2021). Deep Neural Network-Based Prediction of the Risk of Advanced Colorectal Neoplasia. Gut Liver..

[13] Tokutake K., Morelos-Gomez A., Hoshi K.I., Katouda M., Tejima S., Endo M. (2023). Artificial intelligence for the prevention and prediction of colorectal neoplasms. J. Transl. Med..

[14] Li S.J., Sharples L.D., Benton S.C., Blyuss O., Mathews C., Sasieni P., Duffy S.W. (2021). Faecal immunochemical testing in bowel cancer screening: Estimating outcomes for different diagnostic policies. J. Med. Screen..

[15] Yeoh K.G., Ho K.Y., Chiu H.M., Zhu F., Ching J.Y.L., Wu D.C., Matsuda T., Park D.I., Leung W.K., Sollano J. (2011). The Asia-Pacific colorectal screening score: A tool for selecting candidates for screening colonoscopy. Gut.

[16] Liu Y., Liu Z., Liu L., Chen Y., Hu H., Luo L., Zheng M. (2022). Evaluation of the FAST score in colorectal cancer risk stratification. J. Gastroenterol. Hepatol..

[17] Frederiksen B.L., Jørgensen T., Brasso K., Holten I., Osler M. (2010). Socioeconomic position and participation in colorectal cancer screening. Br. J. Cancer.

[18] Palmer C.K., Thomas M.C., Von Wagner C., Raine R. (2014). Reasons for non-uptake in the NHS Bowel Cancer Screening. Br. J. Cancer..

[19] Mansouri D., McMillan D.C., Grant Y., Crighton E.M., Horgan P.G. (2013). Impact of socioeconomic deprivation on outcomes of colorectal cancer patients. Br. J. Surg..

[20] Zhang M., Zhao L., Zhang Y., Jing H., Wei L., Li Z., Zhang H., Zhu S., Zhang S., Zhang X. (2022). Colorectal Cancer Screening in a Population-Based Study. Front. Oncol..

[21] Rahimi F., Rezayatmand R., Shojaeenejad J., Tabesh E., Ravankhah Z., Adibi P. (2023). Costs and outcomes of colorectal cancer screening program in Isfahan, Iran. BMC Health Serv. Res..

[22] Pellat A., Deyra J., Coriat R., Chaussade S. (2018). Results of the national organised colorectal cancer screening program with FIT in Paris. Sci. Rep..

[23] Zahnd W.E., James A.S., Jenkins W.D., Izadi S.R., Fogleman A.J., Steward D.E., Colditz G.A., Goodman M.S. (2018). Rural–urban differences in cancer incidence in the US. Cancer Epidemiol. Biomark. Prev..

[24] Andrilla C.H., Moore T.E., Wong K.M., Evans D.V. (2020). Geographic location and CRC stage at diagnosis. J. Rural. Health.

[25] Choi K.S., Lee H.Y., Jun J.K., Shin A., Park E.C. (2012). Adherence to follow-up after a positive fecal occult blood test in an organized colorectal cancer screening program in Korea, 2004–2008. J. Gastroenterol. Hepatol..

[26] Constantin A., Cazacu I., Ciocârlan M., Constantinescu C., Baltog G., Balahura C., Doraş I., Filip S., Filip G., Panazan I. (2020). Short Term Outcomes of Using Fecal Immunochemical Test for a Pilot Colorectal Cancer Screening Program. Chirurgia.

[27] Rejaibi S., Mchirgui R.M., Ben Mansour N., Barbouch F., Kaddour N., Mrabet A., Aounallah-Skhiri H. (2021). Colorectal cancer mass screening, Tunisia 2019. Tunis Med..

[28] Laiyemo A.O., Doubeni C., Pinsky P.F., Doria-Rose V.P., Bresalier R., Lamerato L.E., Crawford E.D., Kral J.G., Corley D.A., Schoen R.E. (2010). Race and colorectal cancer disparities: Health-care utilization vs different cancer susceptibilities. J. Natl. Cancer Inst..

[29] Gupta S., Kalaivani S., Rajasundaram A., Ameta G.K., Oleiwi A.K., Dugbakie B.N. (2022). AI-based colorectal cancer prediction: Reducing misclassification rates. Cancers.

[30] Mitsala A., Tsalikidis C., Pitiakoudis M., Simopoulos C., Tsaroucha A.K. (2021). Artificial Intelligence in Colorectal Cancer Screening, Diagnosis and Treatment. A New Era. Curr Oncol..

[31] Cubiella J., Digby J., Rodríguez-Alonso L., Vega P., Salve M., Díaz-Ondina M., Strachan J.A., Mowat C., McDonald P.J., Carey F.A. (2017). The fecal hemoglobin concentration, age and sex test score: Development and external validation of a simple prediction tool for colorectal cancer detection in symptomatic patients. Int. J. Cancer.

[32] Cubiella J., Vega P., Salve M., Díaz-Ondina M., Alves M.T., Quintero E., Álvarez-Sánchez V., Fernández-Bañares F., Boadas J., Campo R. (2016). Development and external validation of a faecal immunochemical test-based prediction model for colorectal cancer detection in symptomatic patients. BMC Med.

[33] Yang D.H. (2021). Risk-stratified colorectal cancer screening for optimal use of colonoscopy resources. Korean J. Intern. Med..

[34] Brenne S.S., Ness-Jensen E., Laugsand E.A. (2024). External validation of the colorectal cancer risk score LiFeCRC using food frequency questions in the HUNT study. Int. J. Colorectal. Dis..

[35] Imperiale T.F., Monahan P.O. (2020). Risk Stratification Strategies for Colorectal Cancer Screening: From Logistic Regression to Artificial Intelligence. Gastrointest. Endosc. Clin. N. Am..

[36] Burnett-Hartman A.N., Lee J.K., Demb J., Gupta S. (2021). An Update on Early-Onset Colorectal Cancer. Gastroenterology.

[37] McDowell R., Perrott S., Murchie P., Cardwell C., Hughes C., Samuel L. (2022). Oral antibiotic use and early-onset colorectal cancer. Br. J. Cancer.

[38] Kirkoen B., Berstad P., Hoff G., Høie M., Dovran A., Sagstad S., Tangen T., Kalager M., Skovlund E. (2023). Type and severity of mental illness and participation in CRC screening. Am. J. Prev. Med..

[39] Bhatia D., Sutradhar R., Tinmouth J., Singh S., Lau C., Lipscombe L.L. (2021). Influence of chronic comorbidities on CRC screening participation. Prev. Med..

[40] Goodwin B.C., Myers L., Ireland M.J., March S., Chambers S.K. (2021). Barriers to home bowel cancer screening. Psychooncology.

[41] Law P.J., Timofeeva M., Fernandez-Rozadilla C., Broderick P., Studd J.B., Fernandez-Tajes J., Palles C., Farrington S.M., Svinti V., Ortega J. (2019). Genome-wide association analysis implicates 20 loci in CRC susceptibility. Nat. Genet..

[42] Potter J.D., Slattery M.L., Bostick R.M., Gapstur S.M. (2014). Colorectal cancer: Genes, environment and diet. Nat. Rev. Cancer.

[43] Hull H.R., Sussman J., Starling A., Dunn B., Parkin D.M., Shapiro J.A., Cross A.J., Lin J.S. (2020). External validation of predictive models in colorectal cancer. JCO Clin. Cancer Inform..

[44] Lin D., Wang Y., Wang Y., Li F., Li X., Yan Y., Li Z., Yuan X. (2021). Volatile organic compounds in CRC detection: Potential and challenges. Gut.

[45] Nartowt B., Hart G.R., Mohamed A., Barh D., Godfrey W., Raghavan M.L. (2019). Feature selection in machine learning for cancer screening. Bioinformatics.

[46] Kastrinos F., Kupfer S.S., Gupta S. (2023). Colorectal Cancer Risk Assessment and Precision Approaches to Screening. Gastroenterology.

[47] Peng Y., Mei J., Jiang S., Cheng Y., Deng Y., Zhang W., Zheng S. (2018). Microbiome signatures in CRC patients: Diagnostic and therapeutic implications. Gut Microbes.

[48] Chen H., Zheng X., Zong X., Li Z., Li N., Hur J., Fritz C.D., Chapman W., Nickel K.B., Tipping A. (2021). Metabolic syndrome and risk of early-onset colorectal cancer. Gut.

[49] Cubiella J., Vega P., Salve M., Diaz-Ondina M., Alves M.T., Quintero E., Perez Riquelme F., Carballo F., Ferrandez A., Bujanda L. (2017). Clinical practice guidelines: Colorectal cancer screening. World J. Gastroenterol..

[50] Lee J.K., Liles E.G., Bent S., Levin T.R., Corley D.A. (2018). Comparative effectiveness of screening strategies for colorectal cancer. Ann. Intern. Med..

[51] Lieberman D.A., Rex D.K., Winawer S.J., Giardiello F.M., Johnson D.A., Levin T.R. (2012). Colonoscopy vs FIT for CRC screening: Long-term follow-up. N. Engl. J. Med..

[52] Gupta S., Weiss J., Burke C.A., Dominitz J.A., Green B., Hampel H., Lieu C.H., Robertson D.J., Shapiro J.A., Syngal S. (2020). Patient navigation and CRC screening outcomes. JAMA Intern. Med..

